# Potential Inhibitors of Fascin From A Database of Marine Natural Products: A Virtual Screening and Molecular Dynamics Study

**DOI:** 10.3389/fchem.2021.719949

**Published:** 2021-10-07

**Authors:** Lirui Lin, Kai Lin, Xiaodong Wu, Jia Liu, Yinwei Cheng, Li-Yan Xu, En-Min Li, Geng Dong

**Affiliations:** ^1^ Department of Biochemistry and Molecular Biology, Shantou University Medical College, Shantou, China; ^2^ Medical Informatics Research Center, Shantou University Medical College, Shantou, China; ^3^ Key Laboratory of Molecular Biology in High Cancer Incidence Coastal Area of Guangdong Higher Education Institutes, Shantou University Medical College, Shantou, China; ^4^ Cancer Research Center, Shantou University Medical College, Shantou, China

**Keywords:** marine nature product, fascin, virtual screening, docking, molecular dynamics

## Abstract

Marine nature products are unique compounds that are produced by the marine environment including plants, animals, and microorganisms. The wide diversity of marine natural products have great potential and are versatile in terms of drug discovery. In this paper, we use state-of-the-art computational methods to discover inhibitors from marine natural products to block the function of Fascin, an overexpressed protein in various cancers. First, virtual screening (pharmacophore model and molecular docking) was carried out based on a marine natural products database (12015 molecules) and provided eighteen molecules that could potentially inhibit the function of Fascin. Next, molecular mechanics generalized Born surface area (MM/GBSA) calculations were conducted and indicated that four molecules have higher binding affinities than the inhibitor NP-G2-029, which was validated experimentally. ADMET analyses of pharmacokinetics demonstrated that one of the four molecules does not match the criterion. Finally, ligand Gaussian accelerated molecular dynamics (LiGaMD) simulations were carried out to validate the three inhibitors binding to Fascin stably. In addition, dynamic interactions between protein and ligands were analyzed systematically. Our study will accelerate the development of the cancer drugs targeting Fascin.

## Introduction

With a deeper understanding of the particularity of the marine environment and the diversity of marine biology, researchers have developed many applications based on aquatic and marine resources (Carroll et al., 2021). Extreme conditions in the ocean in terms of temperature, salinity, pressure, and illumination promote marine organisms to evolve and create a unique system with different processes of absorption and metabolism (Montaser and Luesch, 2011). In the metabolism of marine organisms, enormous and innovative marine natural products (MNPs) are produced, and those products can be exploited to develop new functional materials and drugs (Barbosa and Roque, 2019). In recent years, many new compounds have been discovered from marine life, which have also benefited from the rapid development of technology (Hu et al., 2011; Hu et al., 2015; Greco and Cinquegrani, 2016; Ruiz et al., 2016; Blunt et al., 2017; Bilal et al., 2018; Blunt et al., 2018). To exploit the data of MNPs for the treatment of diseases conveniently, some databases of MNPs are built for drug screening and other research on ocean resources (Haroun et al., 2019).

One of the applications of MNPs is drug discovery, e.g., drugs for cancer treatment, as tumor metastasis is the main cause of cancer-related deaths (Chen et al., 2010). Cell invasion and migration are essential features of tumor cells and actin cytoskeleton reconstruction triggers the switch of protrusive tissue, e.g., filopodia, lamellipodia, and lamellipodia (Machesky and Li, 2010). Fascin is one of the actin-binding proteins and it is overexpressed in various types of cancer. Fascin plays a key role in the formation of filopodia, which leads to increased cell movability in multiple transformed cells (Conesa-Zamora et al., 2013; Tan et al., 2013). Some studies have indicated that Fascin can be used as a diagnostic marker and therapeutic target for aggressive tumors (Tan et al., 2013; Rodrigues et al., 2017).

**FIGURE 1:**
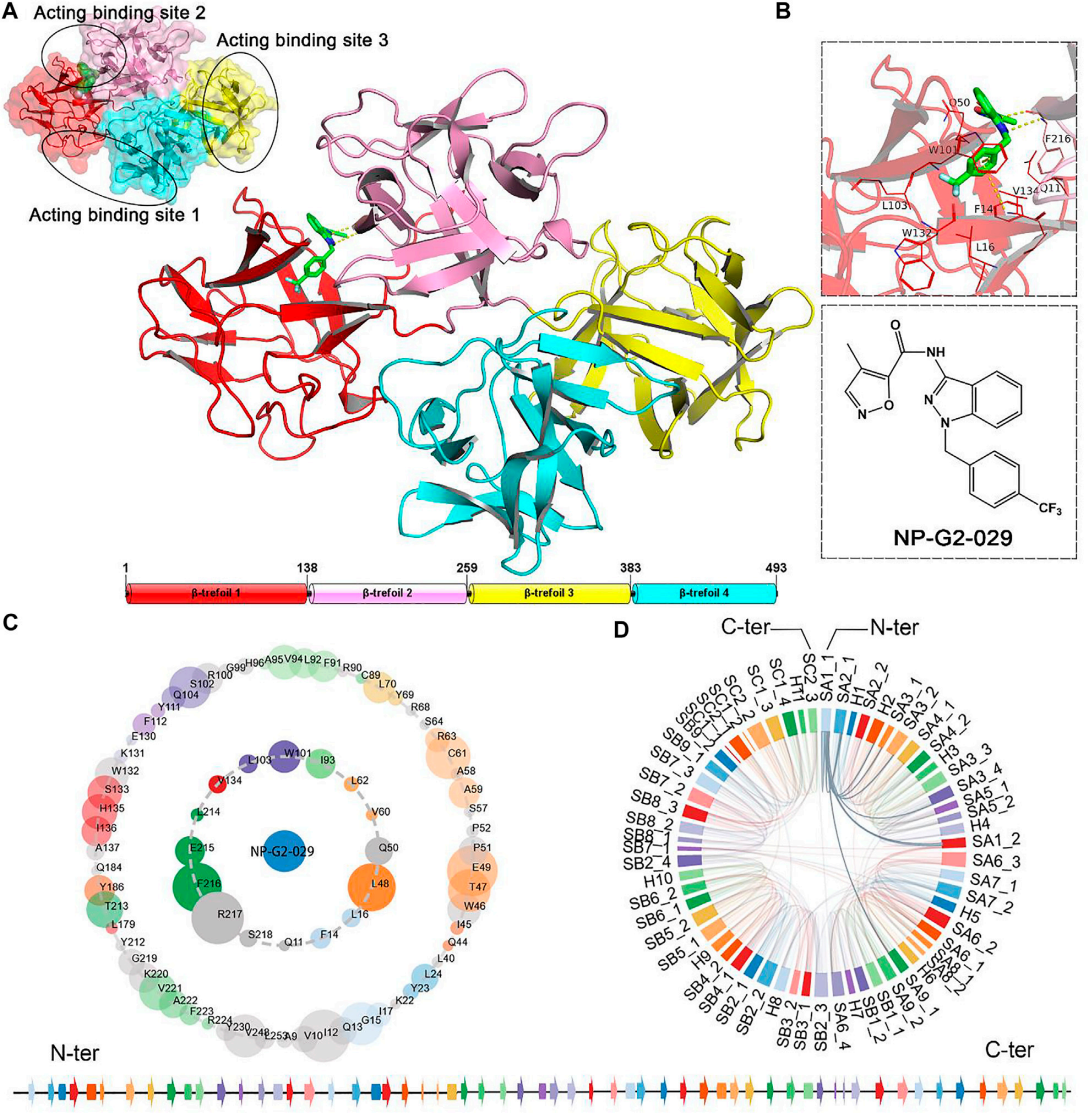
**(A)** The structure of the Fascin-inhibitor complex. Junctions between β-trefoils 1 and 2, β-trefoils 1 and 4 contain two actin-binding sites respectively, and another actin-binding site locates on β-trefoils 3 (Figure 1A). **(B)** Inhibitor NP-G02-029 and the binding pocket. **(C)** Protein−NP-G2-029 interactions are represented by an asteroid plot. The inner ring represents direct interactions. The outer ring represents indirect interactions. The size of the ball is the interaction-number proportion in atomic scale. The colors of residues correspond to their secondary structures. **(D)** Secondary structure connection of Fascin. The bottom panel shows the secondary structure (β-sheets and α helixes) with their respective colors. Arrows stand for β-sheets, rectangles stand for α helix. N-ter, N terminus; C-ter, C terminus. PDB ID: 6B0T. Structure visualized by PyMOL (Rigsby and Parker, 2016).

Fascin was first found as a cross-linking protein in sea urchin (Kane, 1975) and later identified in *Drosophila*, *Xenopus* (Holthuis, Schoonderwoert, and Martens, 1994), mice (Edwards et al., 1995), and human beings (Duh et al., 1994; Yamashiro-Matsumura and Matsumura, 1985). Fascin is one of the components of actin bundles, with 55 k Da and four β-trefoil domains (Figures 1A,B) (Yamashiro-Matsumura and Matsumura, 1985). There are six pairs of two-stranded β-hairpins in each β-trefoil domain with 3-fold symmetry (Murzin, Lesk, and Chothia, 1992; Ponting and Russell, 2000). These four β-trefoils of Fascin form a quadrilateral-like shape and each β-trefoil located in the catercorner (Yamashiro-Matsumura and Matsumura, 1985). Fascin is a monomeric protein and functions by bundling actin filament at its monomeric state. Previous studies have suggested that Fascin has three individual surfaces for its bunding activity to actin, i.e., binding site 1, 2, and 3 (Figure 1A) (Yang et al., 2013). The junction between β-trefoils 1 and 2 of Fascin is suggested to be essential for its actin-bunding activity, which is termed actin-binding site 2. (Figure 1A) (Ono et al., 1997).

To block actin-Fascin interaction and inhibit filament assembly, several small molecule inhibitors have been developed from chemical libraries for biochemical and pathological research (Chen et al., 2010; Huang et al., 2015; Huang et al., 2018). However, the inhibitor exploration for Fascin is still under development, due to the limitation of current inhibitors on efficiency and specificity. NP-G2-029 and NP-G2-044 are two inhibitors targeting Fascin, which show a strong effect, weakening the migration ability of human breast cancer cells (Han et al., 2016; Huang et al., 2018). The IC_50_ values of NP-G2-029 and NP-G2-044 are 0.19 and 0.07 μm in the F-actin-bundling assay. The crystal structure of the Fascin−NP-G2-029 complex was solved by Huang et al. (2018). Six hydrophobic residues surround the benzene ring of NP-G2-029, i.e., Glu11, Phe14, Leu16, Gln50, Trp101, Leu103, Trp132, Val134, and Phe216 (Figure 1B), and the benzene ring also forms edge-to-face pi–pi stacking with Phe14 and Trp101. Two hydrogen-bond interactions are formed between the backbone of Phe216 and the pyrazole and amide groups of NP-G2-029.

The second structure connections of Fascin (Figures 1C,D) show the residues in the binding pocket of NP-G2-029, and the connections of β-sheets and helixes in Fascin. It can be seen from Figure 1D that interactions between secondary structures are complex, indicating that the correlations between those structures are strong. The bottom panel shows the secondary structure (β-sheets and α helixes) with their respective colors (Conducted by Protein Contacts Atlas server) (Kayikci et al., 2018).

In recent years, computer-aided drug discovery (CADD) methods are extensively used for new drug discovery. The pharmacophore model is a ligand-based method to screen lead compounds (Gupta et al., 2019; Wang et al., 2019; Fu et al., 2020; Liu et al., 2020; Liu et al., 2020). It is a rapid and powerful method for the first screening from a large chemical library. The pharmacophore model is often used in combination with structure-based methods, e.g., molecular docking (Saikia and Bordoloi, 2019). Molecular docking programs can be used to predict the bound poses of ligands and to rank them with scoring functions. (Huang and Zou, 2010; Lopez-Vallejo et al., 2011; Garcia-Sosa and Maran, 2021). With CADD approaches, the cost of drug research and development can be reduced markedly (Xiang et al., 2012). These approaches can provide a comprehensive insight into biomolecule mechanisms and improve the effectiveness of the drug development process (Macalino et al., 2015).

It is noteworthy that molecule docking results still need further evaluation and analyses (Rastelli and Pinzi, 2019), and molecular dynamics (MD) simulation is an often-used method to improve the accuracy of molecular docking. Meanwhile, the dynamic properties of proteins can be investigated in depth by MD simulations, which can provide detailed information on the process of ligand binding at an atomic level and this information is significant for drug discovery (De Vivo et al., 2016). Molecular mechanics generalized born surface area (MM/GBSA) is an efficient method for binding free energy calculation, which is used to assess docking poses, determine structural stability and predict binding affinities (Ylilauri and Pentikainen, 2013; Wang et al., 2019). On the other hand, the free energy landscape can be calculated to explore the intermediate states and global minimum of biomolecule (Buckley et al., 2017). However, conformation transition overcoming energy barrier usually needs a millisecond time scale or even longer, depending on the height of the barrier (Miao, Feher, and McCammon, 2015). To overcome this challenge, many enhanced sampling methods have been developed (Bernardi, Melo, and Schulten, 2015). In addition, small molecules have various conformations because of their flexibility in solvent, and the dynamics of small molecules are significant for the induced-fit process (Francis et al., 2019). Thus, exploring the binding state of the inhibitor is important for drug design.

**FIGURE 2:**
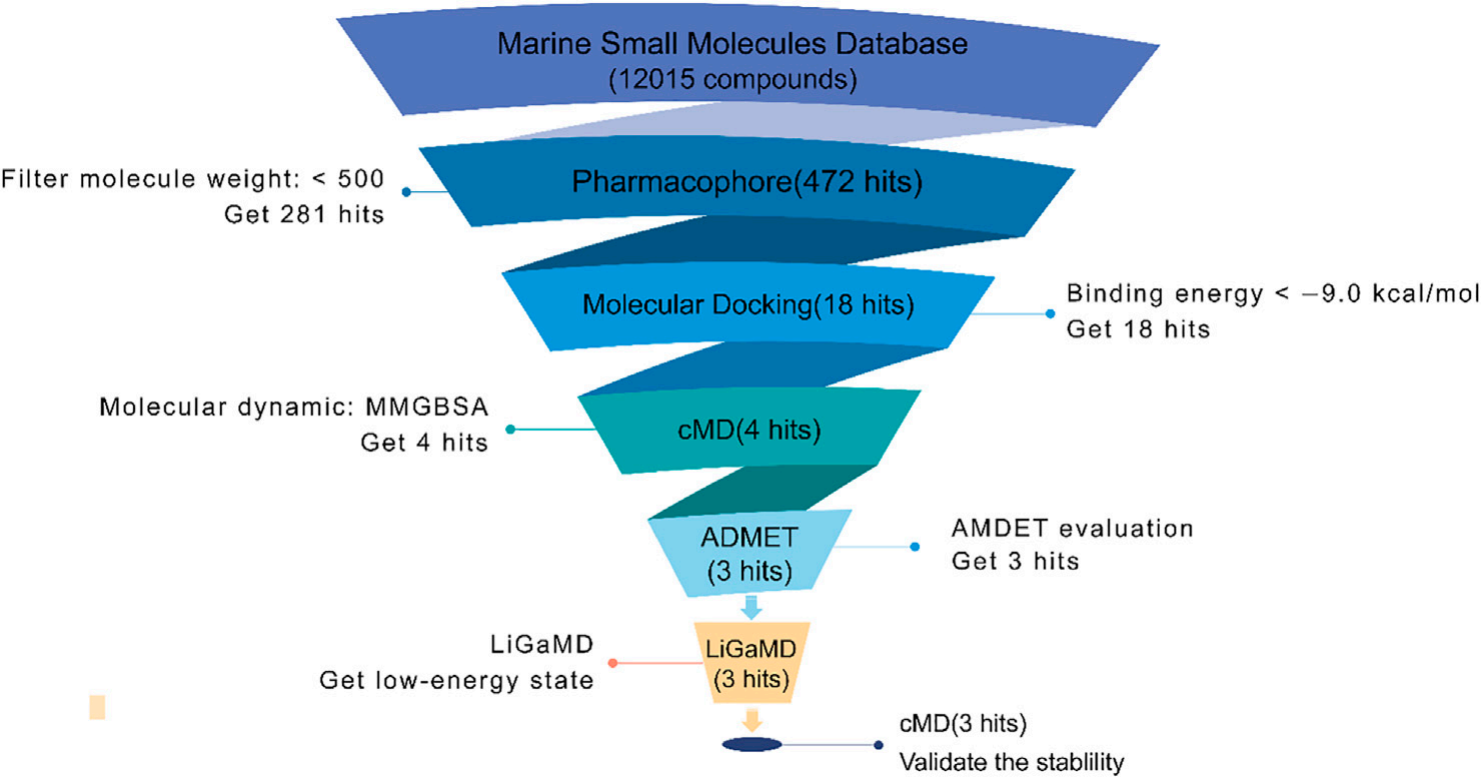
The workflow of inhibitors screening in this study. The pharmacophore model used LigandScout software; docking used the AutoDock Vina module of LigandScout (Nguyen et al., 2020).

In this study, we use several CADD methods to screen small molecules from an MNP library, as indicated by the workflow in Figure 2. First, based on the marine natural products database (12,015 molecules), virtual screening using the pharmacophore model and molecular docking were carried out to discover potential inhibitors of Fascin. Then, the top 18 compounds were selected for further MD simulations, and the binding affinity of each inhibitor was calculated. ADMET predictions were also performed to study pharmacokinetic properties. Furthermore, Ligand Gaussian accelerated Molecular Dynamics (LiGaMD) were carried out on the three potential inhibitors to study low-energy states (Miao, Bhattarai, and Wang, 2020). To validate the low-energy states in LiGaMD, we performed an extended conventional MD. Finally, we analyzed the binding pockets of Fascin with different potential inhibitors.

## Materials and Methods

### Data Preparation

In terms of the target protein, the crystal structure of Fascin was obtained from an online protein database (https://www.rcsb.org), PDB: 6B0T, 2.80 Å resolution (Huang et al., 2018). The crystal structure was solved with its inhibitor NP-G2-029, which was set as an active controlled sample in our study. In addition, NP-G2-044, another effective inhibitor, was used as an active control (Han et al., 2016; Huang et al., 2018). On the other hand, inhibitors NP-G2-112 and NP-G2-113 were used as an inactive control since they do not have any effect on Fascin (Han et al., 2016). Because no complex structures were solved for the NP-G2-044, -112, and -113, complex structures were prepared by molecular docking.

For the ligand database, Marine Natural Products Library (Marvin annotated) series (http://docking.umh.es) was used (Bugni et al., 2008; Encinar et al., 2015; Galiano et al., 2016). OMEGA was used for generating the conformations of all compounds (Hawkins et al., 2010).

### Pharmacophore Model

**FIGURE 3:**
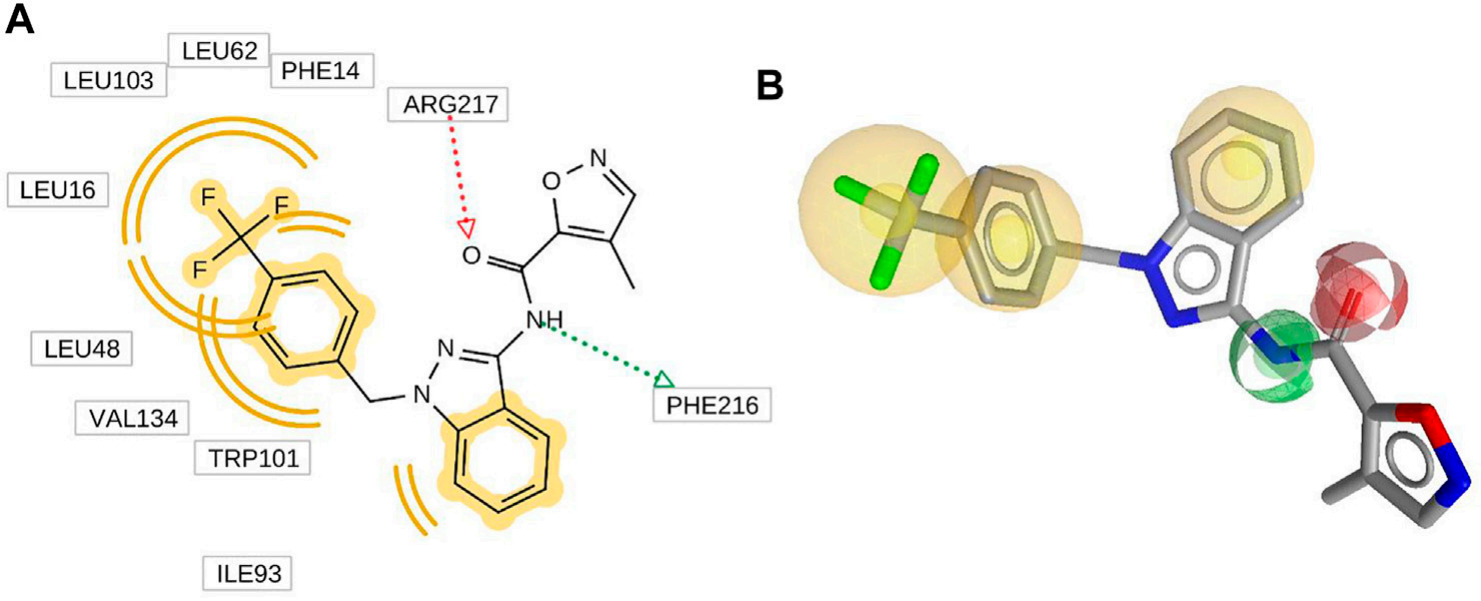
2D **(A)** and 3D **(B)** inhibitor NP-G2-029 with its abstract pharmacophore model generated by LigandScout. Hydrogen bond acceptor (red arrow), hydrogen bond donor (green arrow), hydrophobic interaction, aromatic ring feature interaction (yellow sphere).

Ligand-based pharmacophore modeling is one of the widely used methods in CADD (Leach et al., 2010). In this work, the pharmacophore model was built by LigandScout V_4.4.5_, (Salam, Nuti, and Sherman, 2009; Dixon et al., 2006; Maia et al., 2020). Directed hydrogen-bond interactions, hydrophobic interactions, charge interactions, and steric exclusions were detected directly. In this work, the HypoGen algorithm was used to produce the model, which contains three hydrophobic, one hydrogen-bond donor, and one hydrogen-bond acceptor (Figure 3) (Koes and Camacho, 2011). All features are added as 3D objects. It can be seen from Figure 3A that there are three hydrophobic models for this inhibitor, so the hydrophobic effect is the main pharmacophore feature. In addition, two hydrogen-bond interactions were formed between the inhibitor and Fascin, and inhibitor acted as hydrogen acceptor and donor, respectively.

### Molecular Docking

Molecular docking is a structure based virtual screening method for drug discovery (Liu et al., 2020). It explores small ligand binding to biomacromolecule by searching the possible degrees of freedom of the whole system and finding the global energy minimum. The binding sites of the ligand are evaluated by different score functions. It is widely used for lead screening and optimization (Saikia and Bordoloi, 2019). In this work, the AutoDock Vina module of LigandScout was used for docking (Roy, Srinivasan, and Skolnick, 2015; Nguyen et al., 2020). The scoring function of Vina includes a finite repulsion term, Gaussian steric interaction terms, Piecewise linear hydrophobic, hydrogen-bond interaction terms, etc. (Gaillard, 2018). All docking calculations were performed with default values in LigandScout.

### Molecular Dynamics Simulation

In this paper, we performed molecular dynamic simulations on Fascin with 19 inhibitors. The coordinate of Fascin for all systems was taken from the 2.80 Å crystal structure of the Fascin-NP-G02-029 complex (PDB code: 6B0T) (Huang et al., 2018). Missing residues of structure (fragment 1–6) were modeled by using Chimera (Pettersen et al., 2004). TIP3P water models were used for solvating all systems (Jorgensen et al., 1983) in an octahedral box with a minimum distance of 12 Å from protein structures to box boundary (Gillan, Alfe, and Michaelides, 2016). Each His residue protonation state was identified by the pKa value from PROPKA (Olsson et al., 2011). All of the His residues were protonated at NE2 atoms, except His96, His108, His154, His198, His310 which are assumed to be doubly protonated, and His135 is protonated at ND1 atoms.

For all ligands, AM1-BCC atomic charges were calculated by the antechamber program (Wang and Kollman, 2001) (Jakalian et al., 2000; Jakalian, Jack, and Bayly, 2002). The general AMBER force field (GAFF) and Amber ff14sb force field were used for inhibitors and Fascin, respectively (Wang et al., 2004; Maier et al., 2015). In addition, an optimal amount of counterions was added to generate a neutral system.

The conventional MD simulation of each Fascin-inhibitor system was performed by using Amber 20 (Belfon et al., 2020). Langevin dynamics (Wu and Brooks, 2003) were performed at a constant temperature of 300 K. Collision frequency was set to 2.0 ps^−1^. For NPT ensemble, pressure was kept at 1 atm (Berendsen et al., 1984). Particle mesh Ewald summation was used to handle the long-range electrostatics (Darden, York, and Pedersen, 1993).

For all simulations, we first ran a 5000-step minimization. Then, 20 ps NVT and 20 ps NPT pre-equilibration were carried out with restraints for heavy atoms of the protein. To further equilibrate the system, we ran a 1 ns NPT simulation without any restraints. Finally, 20 ns NPT production simulations were performed and coordinates were printed every 1 ps. For each inhibitor with Fascin, we performed five replicates of production calculations. For all systems, root mean square deviation (RMSD), root mean square fluctuation (RMSF), and radius of gyration (Rg) were calculated by using the cpptraj module in AMBER 20 (Belfon et al., 2020).

### Ligand Gaussian Accelerated Molecular Dynamics

Conformation transition of protein usually happens in a millisecond time scale due to the high energy barrier between different states. Thus, it is hard to capture the most stable state of protein from the whole potential surface. To investigate the conformational changes of Fascin with different inhibitors effectively, we used Li-GaMD (Miao, Feher, and McCammon, 2015; Miao, Bhattarai, and Wang, 2020) for simulation, which is developed from the enhanced sampling method GaMD (Miao, Feher, and McCammon, 2015), LiGaMD can accelerate simulations of the receptor with ligand between binding and unbinding, explore protein conformational transition efficiently.

In a system comprising ligand *L*, protein *P,* and environment *E*, the system comprises *N* atoms with their coordinates $r\equiv \left\{\vec{r_{1}},\ldots ,\vec{r_{N}}\right\}$ and momenta $p\equiv \left\{\vec{p_{1}},\ldots ,\vec{p_{N}}\right\}$ . The system Hamiltonian can be expressed as:

H(r,p)=K(p)+V(r) (1)

where *K(p)* and *V(r)* are the systems kinetic and total potential energies, respectively. Then, the potential energy could be decomposed into the following terms:

$$V\left(r\right)=\;V_{\text{P},\text{b}}\left(r_{P}\right)+\;V_{\text{L},\text{b}}\left(r_{L}\right)+\;V_{\text{E},\text{b}}\left(r_{E}\right)$$

$$+\;V_{\text{PP},nb}\left(r_{P}\right)+\;V_{\text{LL},\text{nb}}\left(r_{PL}\right)+\;V_{\text{EE},\text{nb}}\left(r_{PL}\right)$$

$$+\;V_{\text{PL},\text{nb}}\left(r_{PL}\right)+\;V_{\text{PE},nb}\left(r_{PE}\right)+\;V_{\text{LE},nb}\left(r_{LE}\right)$$ (2)

where *V*_P*,*b_ *, V*_L*,*b,_ and *V*_E*,*b_ are the bonded potential energies in protein *P*, ligand *L,* and environment *E*, respectively. $\;V_{PP,nb}$ , $\;V_{LL,nb}$ , and $\;V_{EE,nb}$ are the nonbonded potential energies. *V*_PL,nb_, *V*_PE,nb_, and *V*_LE,nb_ are the nonbonded interaction energies. Based on classical force fields (Duan et al., 2003; Vanommeslaeghe and MacKerell, 2015), the non-bonded potential energies are usually presented as:

$$\;V_{nb}=\;V_{elec}+\;V_{vdw}$$ (3)

Presumably, ligand binding mainly involves the nonbonded interaction energies of the ligand, $\;V_{\text{L},\text{nb}}\left(\text{r}\right)=\;V_{\text{LL},\text{nb}}\left({\text{r}}_{\text{L}}\right)+\;V_{\text{PL},\text{nb}}\left({\text{r}}_{\text{PL}}\right)+\;V_{\text{LE},\text{nb}}\left({\text{r}}_{\text{LE}}\right)$ . LiGaMD adds a boost potential selectively to the ligand non-bonded potential energy according to the GaMD algorithm:

$$\Delta V_{L,nb}\left(r\right)=\{\begin{array}{c} \frac{1}{2}\;k_{L,nb}{\left(E_{L,nb}-V_{L,nb}\left(r\right)\right)}^{2},\;\;V_{L,nb}\left(r\right)<E_{L,nb}\;\;\\ 0,\;V_{L,nb}\left(r\right)<E_{L,nb}\; \end{array}$$ (4)

where $E_{\text{L},\text{nb}}$ is the threshold energy for applying boost potential and $k_{\text{L},\text{nb}}$ is the harmonic constant. These parameters in LiGaMD are derived similarly as in the GaMD algorithm (Miao, Feher, and McCammon, 2015).

In addition to optional boosting non-bonded potential energy term of ligand, a second boost potential can be added on protein to explore protein conformational changes. The second boost potential is calculated using the total system potential energy as:

$$\Delta V_{D}\left(r\right)=\{\begin{array}{c} \frac{1}{2}\;k_{D}{\left(E_{D}-V_{D}\left(r\right)\right)}^{2},\;\;V_{D}\left(r\right)<E_{D}\;\;\\ 0,\;\;\;\;\;\;\;\;\;\;\;\;\;\;\;\;\;\;\;\;\;\;\;\;\;\;\;\;\;\;\;\;\;\;\;\;\;\;\;\;\;\;\;\;\;\;\;V_{D}\left(r\right)<E_{D}\; \end{array}$$ (5)

where $V_{D}$ is the total potential energy without the nonbonded potential energy of ligand, $E_{D}$ is the threshold energy for applying the second boost potential and $k_{D}$ is the harmonic constant. In this study, we applied dual-boost LiGaMD and total boost potential $\Delta V\left(r\right)=\Delta V_{L,nb}\left(r\right)+\Delta V_{D}\left(r\right)$ = Δ*V*_L,nb_(r) + Δ*V*_D_(r). For the analysis of the results, we used the PyReweighting program to calculate the free energy surface with different collective variables (Miao et al., 2014).

### Binding Affinity Calculation With MM/GBSA

In order to calculate the binding free energies for different potential inhibitors, we used molecular mechanics MM/GBSA methods. It is an end-point based free energy calculation method, i.e., the binding free energy is calculated by

ΔG_bind_ = G_RL_ – G_R_ – G_L_(6) (6)

where G_L_, G_R,_ and G_L_ represent the free energy of the complex and the receptor and ligand, respectively. Each free energy is calculated with

$$\text{G}=\left({\text{E}}_{\text{bond}}\right)+\left({\text{E}}_{\text{ele}}\right)+\left({\text{E}}_{\text{vdW}}\right)+\left({\text{E}}_{\text{pol}}\right)+\left({\text{E}}_{\text{np}}\right)-\text{TS}$$ (7)

where E_bond_ is the energy of covalent interactions, E_ele_ is the electrostatic potential, E_vdW_ is the energy of van der Waals interactions, and G_pol_ and G_np_ are the polar and nonpolar contributions. The conformational entropy contribution (-TS) is estimated by normal-mode analysis (Srinivasan et al., 1998), but it is usually neglected from consideration due to its high computational cost and low prediction accuracy (Hou and Yu, 2007; Sun et al., 2018). In this work, MMPBSA. py (Miller et al., 2012) module in Amber20 (Belfon et al., 2020) was used to calculate the MM/GBSA for each system based on the last 2,500 frames extracted from the 20 ns conventional MD trajectory.

### Pharmacokinetics Evaluation

ADMET evaluation is a comprehensive study of drug absorption, distribution, metabolism, excretion, and toxicity properties (Acuna, Hopper, and Yoder, 2020). Evaluation of ADMET properties at the early stage of drug development can significantly improve the success rate of drug discovery. It is used to efficiently and accurately calculate the physicochemical properties, toxicity information, and pharmacokinetic properties of candidate drug molecules, provide the basis for prediction and improve the interpretability of structure and drug credibility (Wenzel, Matter, and Schmidt, 2019). The small molecule hits were predicted by ADMET based on the Swissadme server (http://www.swissadme.ch/) (Daina, Michielin, and Zoete, 2017), and LogP and TPSA were pointed out as the main reference indexes of the results.

Log P refers to the equilibrium distribution of the undissociated molecules in the oil and water phases, which is an important indicator in the passage of compounds through biofilm. TPSA refers to the topological polar surface area. TPSA <60 indicates that it has good membrane permeability and is completely absorbed. 60 < TPSA <140 indicates the molecular permeability decreases with the increase of polar surface area. TPSA >140 indicates poor permeability of the molecule. Lipid solubility is an important parameter of small molecules in pharmaceutical chemistry. Log P is the logarithm of the oil-water partition coefficient P of the compound, which refers to the equilibrium of the distribution of the undissociated molecules in the oil phase and water phase. When oral drugs are permeated by passive diffusion, logP in the 0–3 range is the best. High logP compounds have poor water solubility and low logP compounds have poor lipid permeability.

## Results and Discussion

### Pharmacophore Model

In this work, the pharmacophore model with three hydrophobic, one hydrogen-bond donor, and one hydrogen-bond acceptor (Figure 3) was used for virtual screening. First, the MNPs database (14,064 compounds) was processed by Openbabel V_2.4.1_ for 3D structure generation, hydrogenation, and charge processing operation (O'Boyle et al., 2011), and 12,015 compounds were generated. Then, the pharmacophore virtual screening was performed on the 12,015 compounds (Figure 3). In total, 472 compounds with high fitness were found. Finally, compounds that have a molecular weight larger than 500 were removed with the *Filter* module, which provided 281 results for further study.

### Molecular Docking

**FIGURE 4:**
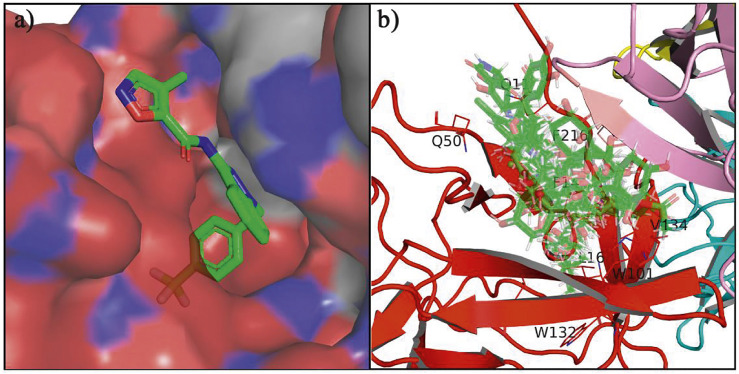
**(A)** The binding pocket of Fascin with inhibitor NP-G2-029; **(B)** The molecular docking results for 18 small-molecules.

In the crystal structure, inhibitor NP-G2-029 resides in the pocket formed by the residues located in the junction of domains 1 and 2 (Figure 4A). The surface volume of the active site inherent is 1130 A^3^ calculated by the Proteins Plus server (Schoning-Stierand et al., 2020). To rank the 281 ligands from the screening based on the Pharmacophore model, molecular docking was performed with the AutoDock Vina module of the Ligandscout program (Wolber and Langer, 2005). The top 18 ligands with binding energy ≤ −9 kcal/mol were selected as the potential inhibitors for further calculations. The molecular structures and molecular binding energy are shown in Table 1 (For convenience, we have also provided the ZNIC ID for those MNPs).

**TABLE 1:** Data collection of potential inhibitors for Fascin by molecular docking. Unit: kcal/mol.

No./Compound	Library ID	Structure	Molecular weight	Binding affinity
01_a_ NP-G2-029 C_20_H_15_F_3_N_4_O_2_	No data	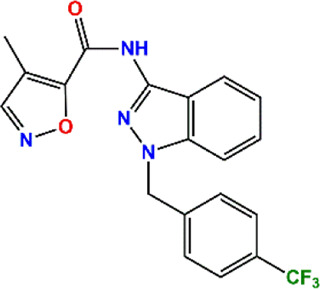	400.36	−10.80
02 C_25_H_36_O_6_	ZINC000238749885	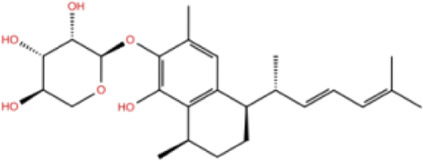	432.56	−10.50
03 C_28_H_22_O_7_	ZINC000014693073	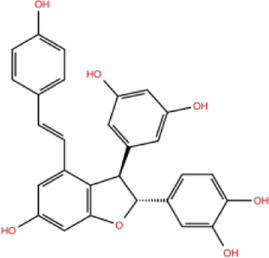	470.48	−9.90
04 C_27_H_46_O_5_	ZINC000044387599	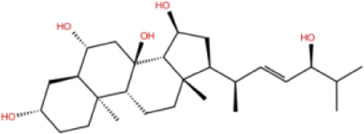	450.66	−9.70
05 C_25_H_35_NO_5_	ZINC000014714664	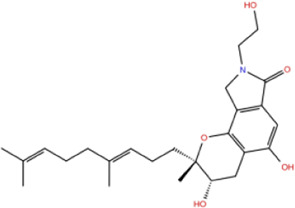	429.56	−9.60
06 C_25_H_34_O_6_	ZINC000238761262	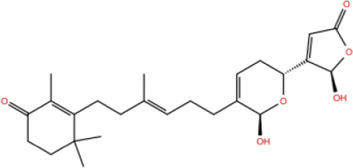	430.54	−9.50
07 C_25_H_38_O_6_	ZINC000040915743	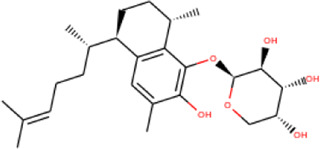	434.57	−9.40
08 C_27_H_34_N_2_O_5_	ZINC000042851223	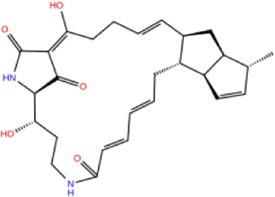	466.58	−9.40
09 C_25_H_25_NO_6_Cl	No data	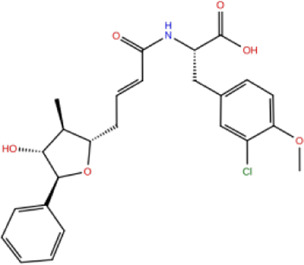	470.93	−9.30
10 C_29_H_50_O_6_	ZINC000255214715	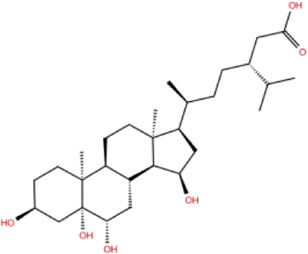	494.71	−9.30
11 C_28_H_40_O_7_	ZINC000042888842	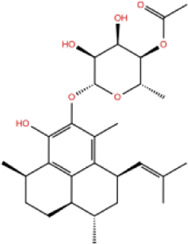	488.62	−9.20
12 C_20_H_28_O_4_	ZINC000005890667	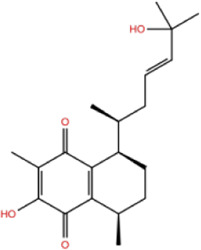	332.44	−9.10
13 C_28_H_40_O_3_	ZINC000014767734	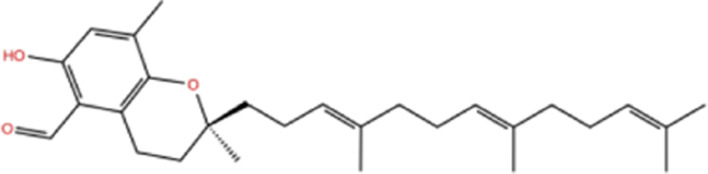	424.62	−9.10
14 C_27_H_48_O_4_	ZINC000137671675	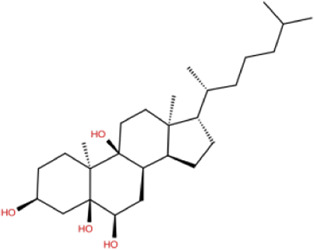	436.68	−9.10
15 C_28_H_50_O_4_	ZINC000137547990	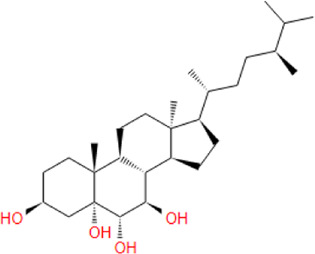	450.70	−9.10
16 C_30_H_52_O_4_	No data	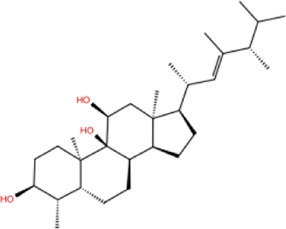	476.74	−9.10
17 C_24_H_20_Cl_2_N_2_O_4_	ZINC000085599962	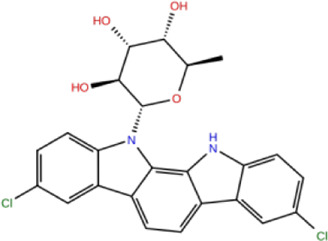	471.34	−9.10
18 C_27_H_39_N_3_O_2_	No data	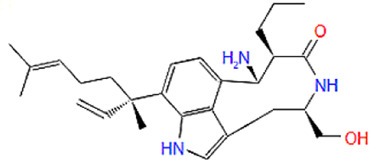	437.63	−9.00
19 C_28_H_48_O_6_	ZINC000044387005	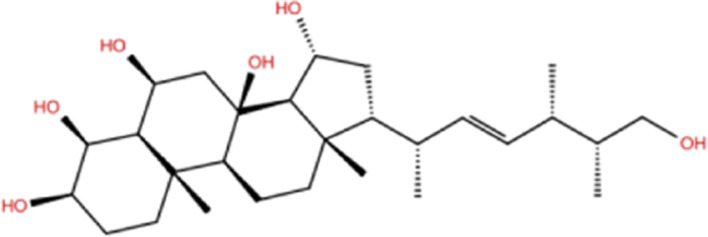	480.69	−9.00

a Active controlled indicator.

As is shown in Figure 4B, all the 18 small molecules that are embedded in the binding pocket are approximately as same as NP-G2-029. The residues of Fascin involved in the binding pocket are mainly Leu48, Glu49, Gln50, Ile93, Trp101, Val 103, Glu215, Phe216, and Arg217. All the detailed interactions between ligands and proteins are shown in Supplementary Figure S1.

### Conventional Molecular Dynamics for Fascin-Inhibitor Complex

**FIGURE 5:**
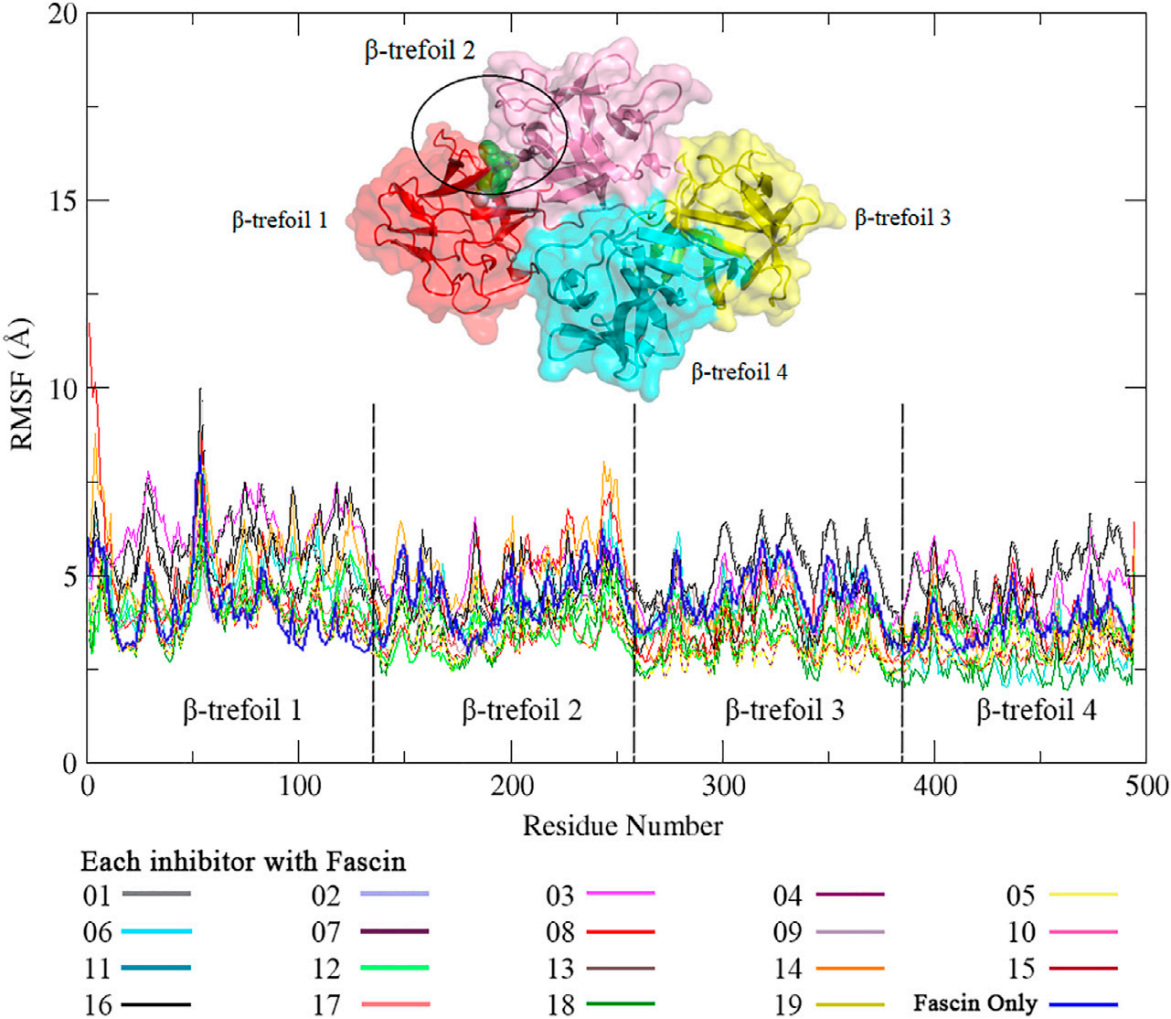
The RMSF of residues in complex with NP-G2-029 and 18 inhibitors in conventional MD simulations. Different color lines stand for the different inhibitors.

To find a better inhibitor than NP-G2-029, conventional MD dynamics were carried for the Fascin with 18 inhibitors from AutoDock Vina. For each system, we run 20 ns × 5 replicates. RMSDs for all systems indicate that all simulations are converged (Supplementary Figure S2). It can be seen from RMSF data (Figure5) that the binding sites of inhibitor in Fascin-inhibitor complexes are more dynamic with a range of 3–10 Å in β-trefoil 1, whereas other regions are relatively rigid, compared to Fascin without inhibitor (Blue line in Figure 5). These findings are consistent with a study by Huang et al. (2018). Overall, the inhibitors affect the RMSF of Fascin significantly.

### Binding Free Energy by MM/GBSA

To improve the accuracy of ranking in molecular docking, we calculated the binding affinities of inhibitors in each complex using MM/GBSA with conventional MD trajectories. Binding free energy results were obtained based on the five replicate (20 ns × 5 replicas) simulations (Table 2). For inhibitor NP-G2-029 with Fascin, the calculated binding affinity is −41 kcal/mol, indicating the two objectives are favorable for binding, which is consistent with experimental data that NP-G2-029 inhibits Fascin (Huang et al., 2018). For the other active inhibitor NP-G2-044, the binding affinity is −42 kcal/mol (Supplementary Table S2). For the inactive inhibitors NP-G2-112 and NP-G2-113, they are −38 and −35 kcal/mol, respectively. Thus, −41 kcal/mol was used as a threshold value, i.e., ligands with binding affinity larger than −40 kcal/mol are thought of as potential inhibitors. It can be found from Table 2, No. 07, 13, 15, 18 inhibitors have qualified binding affinities to Fascin with binding free energies of −41, −47, −41, −44 kcal/mol, respectively.

**TABLE 2:** Binding affinity for each inhibitor by MM/GBSA (Unit: kcal/mol. Potential inhibitors those meet the criterion are highlighted as bold values.)

Receptor	Ligand No.	△E_vdW_	△E_ele_	△E_Polar_	△E_non-polar_	ΔG_bind_	SD
Fascin	01(NP-G02-029)	−50.42	−20.92	35.29	−4.93	−40.97	0.28
Fascin	02	−43.71	−19.74	34.51	−4.98	−33.91	3.54
Fascin	03	−44.63	−28.99	51.13	−5.18	−27.66	3.07
Fascin	04	−47.44	−15.72	33.96	−5.04	−34.23	3.42
Fascin	05	−47.86	−11.60	27.43	−5.38	−37.41	2.51
Fascin	06	−45.80	−11.71	31.96	−5.03	−30.58	3.79
Fascin	**07**	−**52.78**	−**10.03**	**27.01**	−**5.35**	−**41.14**	**2.53**
Fascin	08	−42.05	−29.61	42.83	−4.60	−33.41	2.66
Fascin	09	−45.42	−16.57	36.71	−5.24	−30.52	2.62
Fascin	10	−40.84	−17.14	32.68	−4.80	−30.10	2.65
Fascin	11	−43.02	−19.21	33.22	−5.21	−34.22	3.07
Fascin	12	−38.20	−6.21	21.80	−4.28	−26.89	1.79
Fascin	**13**	−**58.00**	−**15.99**	**32.75**	−**5.97**	−**47.20**	**3.94**
Fascin	14	−47.53	−8.89	25.55	−5.01	−35.88	2.09
Fascin	**15**	−**49.60**	−**18.33**	**32.55**	−**5.26**	−**40.64**	**0.69**
Fascin	16	−46.01	−7.04	21.67	−5.06	−36.44	2.42
Fascin	17	−42.97	−32.28	45.93	−4.74	−34.06	2.59
Fascin	**18**	−**56.03**	−**12.02**	**29.80**	−**5.55**	−**43.79**	**2.56**
Fascin	19	−47.41	−9.60	31.15	−5.18	−31.04	2.86

### Pharmacokinetics Evaluation

Pharmacokinetics prediction was performed for the 19 compounds (including NP-G2-029) on the ADMETlab server (Dong et al., 2018), which is based on a comprehensive database that includes 288,967 entries (Ferreira and Andricopulo, 2019). There are four function modules for drug-likeness analysis, ADME endpoint prediction, systematic evaluation, and similarity searching, these results give an overall understanding of compounds and can check the rapid screening process.

For NP-G02-029 and the 18 small molecules, we perform ADMET assessments, which include Lipid solubility (Dong et al., 2018; Ferreira and Andricopulo, 2019). Lipid solubility is an important parameter for small molecules in pharmaceutical chemistry (Williams et al., 2013). When oral drugs are permeated by passive diffusion, the logP 0–3 range is the best. High logP compounds have poor water solubility, low logP compounds have poor lipid permeability. TPSA <60 denotes good membrane permeability and is completely absorbed. 60 < TPSA <140 denotes that the molecular permeability decreases with the increase of polar surface area. TPSA >140 denotes the poor permeability of the molecule.

**FIGURE 6:**
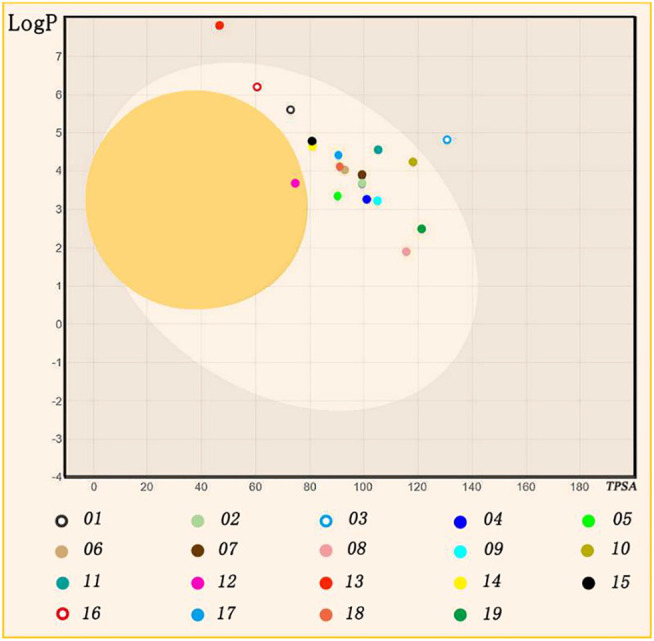
ADME evaluation result. Different color circles stand for each inhibitor.

ADME results in Figure 6 show that TPSA of No. 02, 04, 05, 06, 07, 09, 10, 11, 12, 14, 15, 17, 18, 19 are in range of 60–120, and whose logP are in a range of 3–5. Notable, No.13 inhibitor is out of 60–120 TPSA and 3–5 logP, therefore, we exclude No.13 inhibitor for the next assessment.

**FIGURE 7:**
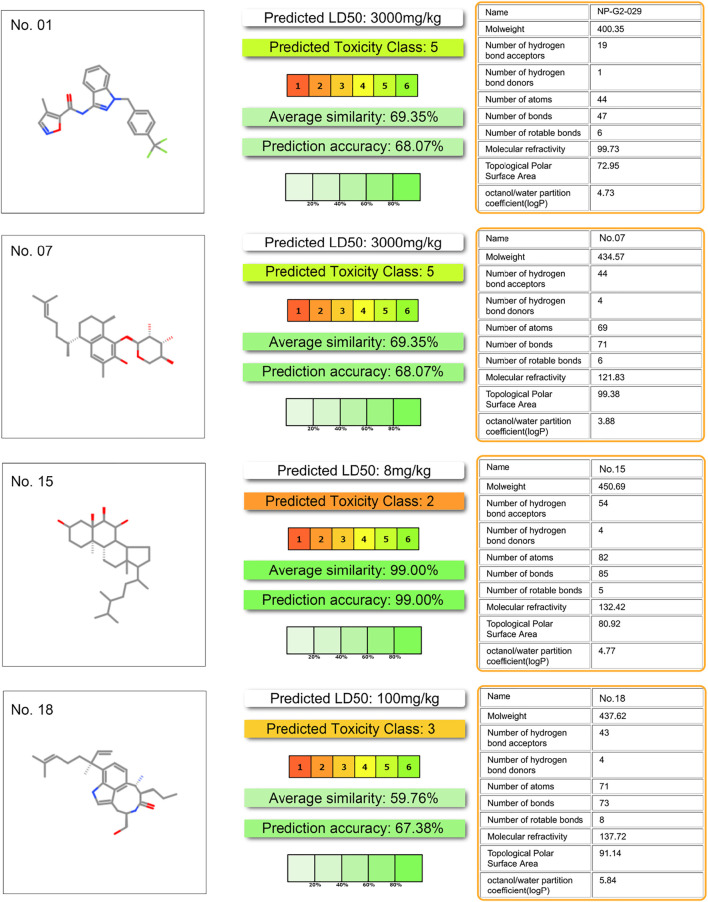
Toxicity evaluation result. Evaluation processed by ProTox-II server (https://tox-new.charite.de/) (Banerjee et al., 2018).

Toxicity predictions are performed on the potential inhibitor No. 07, 15, 18, and the NP-G2-029. Data in Figure 7 shows that the toxicity score of NP-G2-029 is 5, the toxicity scores of inhibitor No. 07, 15, 18 are 5, 2, 3 respectively, whose are lower than NP-G2-029, signify that the potentials inhibitor are less toxic than NP-G2-029.

### Ligand GAMD Simulation

To confirm whether the docking pose of compounds 07, 15, and 18 are stable in the pocket of Fascin, we performed LiGaMD simulations. The boost potential added in LiGaMD simulations is according to Gaussian distribution, accurate reweighting and recovery of the original biomolecular free energy landscapes can be achieved by using cumulant expansion to the second order.

**FIGURE 8:**
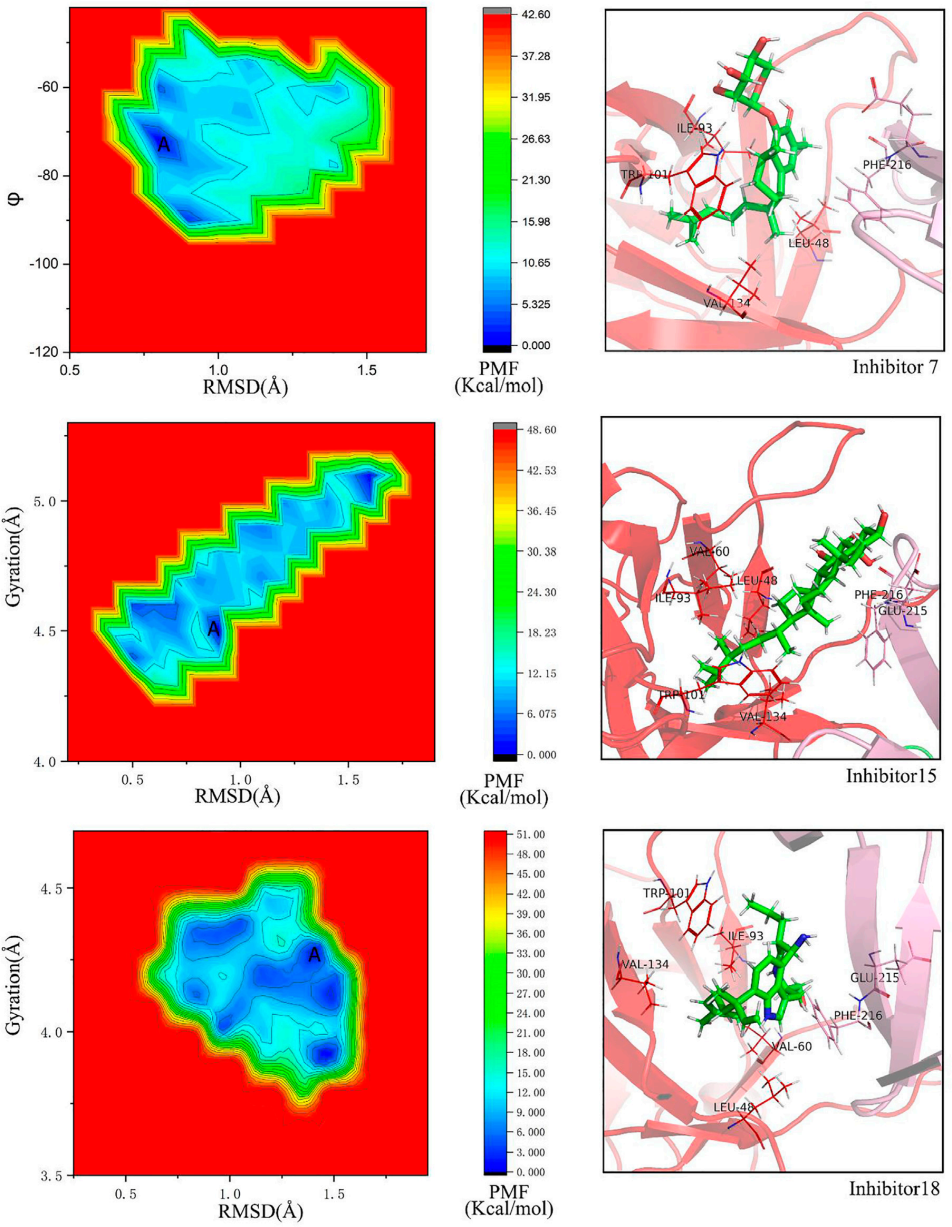
Free energy landscapes and low-energy conformational states of Fascin with inhibitors, whose was modeled with the GAFF force field by using LiGaMD simulation: **(A)** PMF profile of inhibitor No. 07, collective variables (CVs) are backbone dihedrals (*φ*) and RMSD of inhibitor No.07. **(B)** PMF profiles of inhibitor No. 15, CVs are the radius of gyration and RMSD. **(C)** PMF profiles of inhibitor No. 18, CVs are the same with inhibitor No.15.

For No. 07 inhibitor (Figure 8A), 2D PMF with backbone dihedrals (*φ*) and RMSD is calculated by reweighting 100 ns LiGaMD simulations. One low-energy state (labeled as A) can be found from the potential surface. The binding pocket in this state includes Ile93, Trp101, Val134, Phe216, Leu48, Val60, Phe14, Leu103, Leu16, and they contribute the binding free energy much according to the energy decomposition in MM/GBSA (Figure 10).

For No. 15 inhibitor (Figure 8B) 2D PMF with the radius of gyration of protein and RMSD. The state with the lowest energy is shown in Figure 8B. For this inhibitor, the binding pocket is slightly modulated due to ligand binding. The pocket consists of Glu215, Val134, Phe216, Arg217, Leu48, Ile93, Val60, Trp101, Phe14, and Leu16.

For the No.18 inhibitor (Figure 8C), the 2D PMF was plotted with the same collective variables as inhibitor No. 15. Again, the binding pocket modulates slightly, including Ile93, Phe216, Trp101, Val134, Leu48, Glu215, Val60, Phe14, and Leu103.

In addition, for binding poses of the ligands, AutoDock Vina gives almost the same pose as LiGaMD simulations in this study.

### Extended Conventional MD From Low-Energy States

**FIGURE 9:**
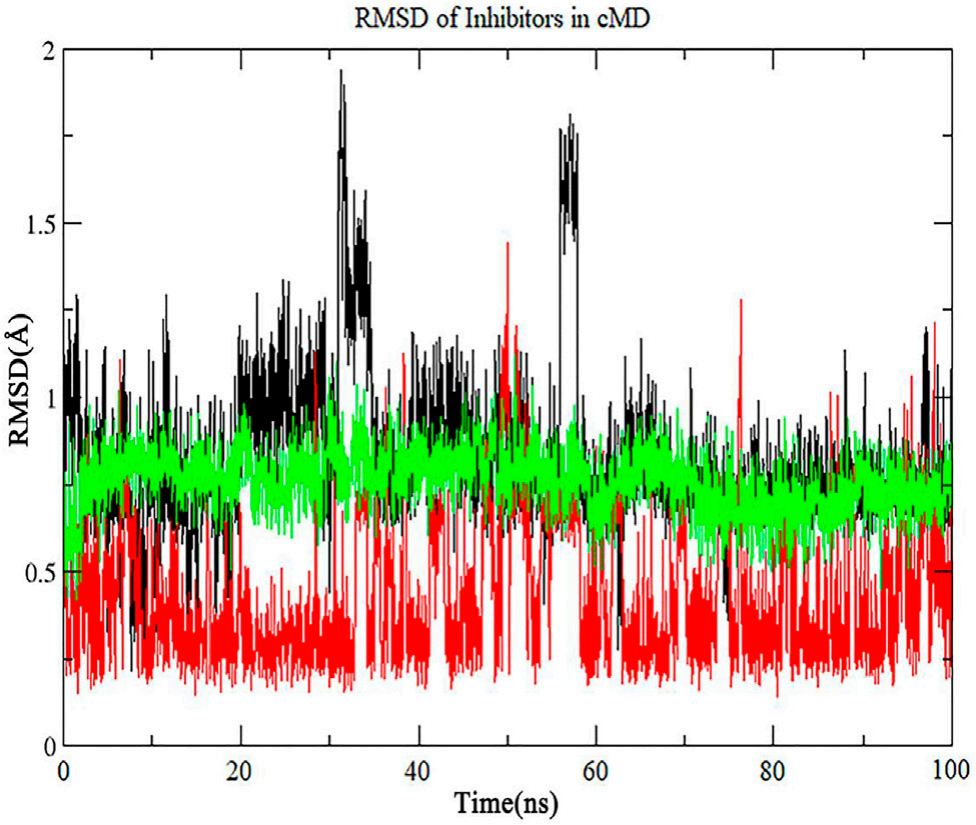
The RMSD of inhibitor No. 07, 15, 18 in extended conventional MD simulation. The line with black, red, green color is inhibitor No. 07, 15, 18 respectively.

To confirm that whether the three inhibitors reside in the binding pocket of Fascin in LiGaMD, we ran 100 ns conventional MD simulations that start from the structures at A position in Figure 8. As is shown in Figure 9, RMSD values for the three potential inhibitors are mainly lower than 1 Å, indicating that all of them stay at the binding position. On the other hand, our results indicate that the docking structures can be trusted for this system.

**FIGURE 10:**
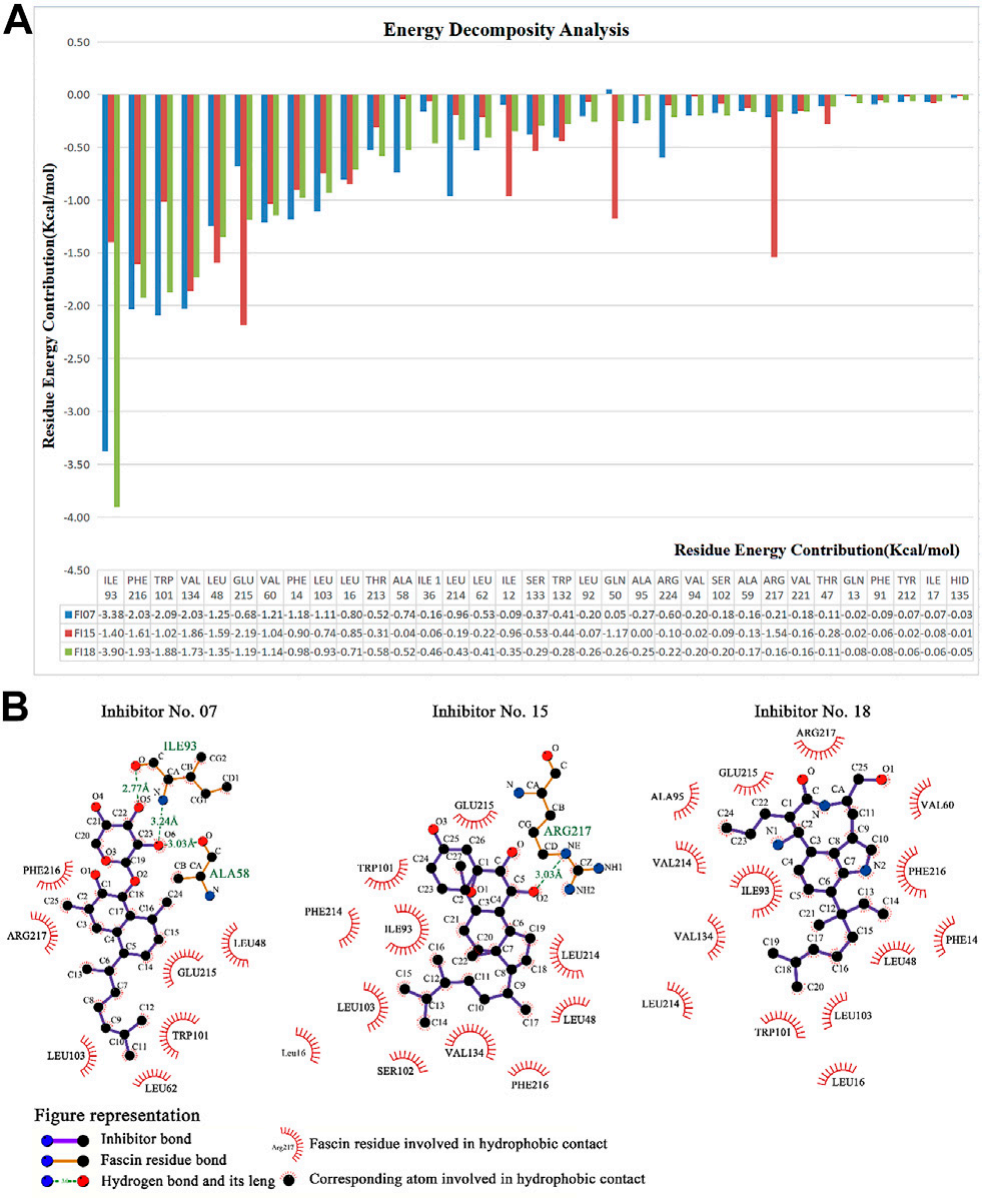
Energy decomposition analysis and interaction networks between Fascin and inhibitors No. 07, 15, and 18. Interaction networks plotted by LIGPLOT Software (Laskowski and Thornton, 1995).

Finally, we run the binding affinity and binding energy decomposition analysis for the three systems (Figure 10A). The binding affinities of inhibitor No. 07, 15, 18 are −42, −45, and −41 kcal/mol, respectively. Our results indicate the three compounds can bind to Fascin as well as NP-G2-029. The crucial residues contributed to the binding affinity of the inhibitor and are mainly in the binding pocket, as shown in Figure 10B. It can be seen from the interaction network that the hydrophobic interactions have a large contribution for binding, i.e., 7 hydrophobic interactions for ligand No. 07, 11 hydrophobic interactions for ligand No. 15, and 24 hydrophobic interactions for ligand No. 18. In addition, 2 hydrogen bonds are formed for ligand No. 07 and one hydrogen bond is formed for ligand No. 15.

## Conclusion

Fascin is overexpressed in many cancers, e.g., esophageal cancer. In this paper, we performed CADD methods to predict the potential inhibitors for Fascin from a library of marine natural products including 14,064 compounds, viz. pharmacophore model, molecular docking, molecular dynamics, MM/GBSA, and predictions of absorption, distribution, metabolism, excretion and toxicity properties (AMDET).

First, we built the pharmacophore model for the inhibitor NP-G02-029, which was confirmed experimentally (Huang et al., 2018). With the pharmacophore model, we achieved 472 results. In addition, compounds that have a molecular weight larger than 500 were kicked out, which gives 281 hits for further study. Next, molecular docking was carried out to rank all the 281 hits. The top 18 inhibitors with binding affinity larger than 9 kcal/mol were selected for further study.

To accurately assess the binding affinity, MM/GBSA calculations are performed for the 19 compounds (including NP-G02-029). Four compounds (No. 07, 13, 15, and 18) were found to have larger affinities to Fascin than NP-G02-029 and were deemed potential inhibitors.

To predict the AMDET, we used the web server ADMETlab server (Dong et al., 2018) and ProTox-II server (Banerjee et al., 2018). AMDET results indicate that compound No.13 does not satisfy the criteria. Thus, three compounds (No. 07, 15, and 18) potentially inhibit the function of Fascin.

Finally, we ran LiGaMD and other conventional MD simulations to confirm whether the three potential inhibitors reside in the binding site or not. Our results demonstrate that all of them stay at the binding site stably.

Thus, we predict three potential inhibitors for Fascin from marine natural products in this investigation. These inhibitors could have higher binding affinities than the one (NP-G02-029) found in the previous study (Huang et al., 2018), and block the function of Fascin. All the computational methods used in this study could accelerate drug discovery dramatically.

## Data Availability

The raw data supporting the conclusions of this article will be made available by the authors, without undue reservation.
