# Supplementary material for: Potential Inhibitors of Fascin From A Database of Marine Natural Products: A Virtual Screening and Molecular Dynamics Study
Source: Front Chem. 2021 Oct 7;9:719949. doi: 10.3389/fchem.2021.719949 (PMC8529705; doi:10.3389/fchem.2021.719949)
Supplement: Supplementary file 1 [file DataSheet2.PDF]

Supporting Information

Potential Inhibitors of Fascin from A Database of Marine Natural Products: A Virtual Screening and Molecular Dynamics Study

Lirui Lin[1,2], Kai Lin[1,2], Xiaodong Wu[1], Jia Liu[1], Yinwei Cheng[3,4], Li-Yan Xu[*3,4], En-Min Li[*1,3], Geng Dong[*1,2]

[1] Department of Biochemistry and Molecular Biology, Shantou University Medical College, Shantou, 515041, PR China

[2] Medical Informatics Research Center, Shantou University Medical College, Shantou, 515041, PR China

[3] Key Laboratory of Molecular Biology in High Cancer Incidence Coastal Area of Guangdong Higher Education Institutes, Shantou University Medical College, Shantou, 515041, PR China

[4] Cancer Research Center, Shantou University Medical College, Shantou, 515041, PR China

Correspondence to lyxu@stu.edu.cn,
or En-Min Li, E-mail: nmli@stu.edu.cn,
or Geng Dong, E-mail: gdong@stu.edu.cn

TABLE S1 Computational binding affinities of active and inactive ligands based on MM/GBSA in 5 replica MD simulations, Unit: Kcal/mol

**Item**	**Ligand**	**1st MD**	**2nd MD**	**3rd MD**	**4th MD**	**5th MD**	**Average**
Active	NP-G2-044	–41.18	–41.71	–41.11	–41.94	–41.32	–41.45
Inactive	NP-G2-112	–38.18	–38.88	–38.49	–38.46	–38.27	–38.45
Inactive	NP-G2-113	–35.95	–35.76	–35.66	–35.47	–35.15	–35.60

TABLE S2 ZINC IDs and the 2D structures of active and inactive ligands

**Compound/No.**	**Structure**
NP-G2-044<br>$C_{21}H_{16}F_3N_3O_2$	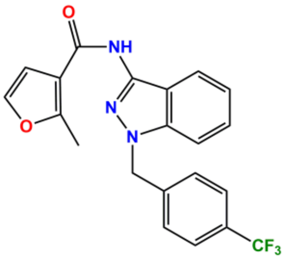
NP-G2-112<br>$C_{20}H_{16}FN_3O_2$	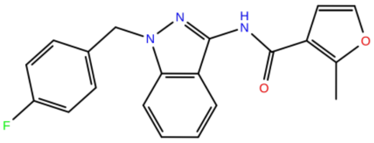
NP-G2-113<br>$C_{19}H_{16}FN_4SO$	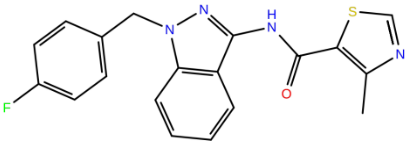

TABLE S3 Binding affinities for small-molecule No. 20 to 30 in molecule docking. Unit: kcal/mol

No.	Zinc Number	Molecule weight	Chemical Structure	Binding affinity
No. 20	ZINC000044387005	480.68	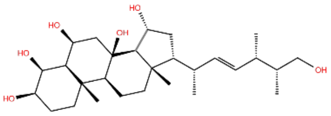	-33.35
No. 21	ZINC000033977462	302.45	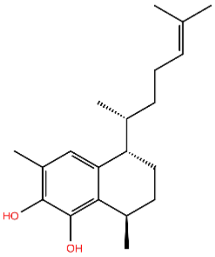	-30.68
No. 22	ZINC000003874163	416.55	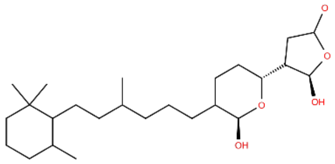	-28.33

No. 23	ZINC000038299972	430.62	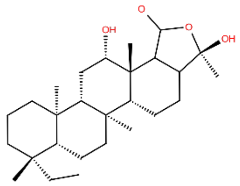	-31.74
No. 24	ZINC000137551534	458.72	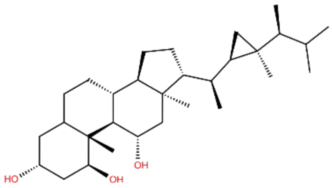	-36.54
No. 25	ZINC000255258130	428.65	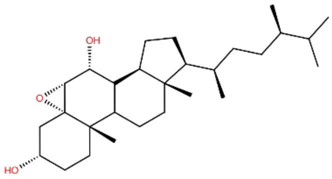	-36.88
No. 26	ZINC000140370169	446.58	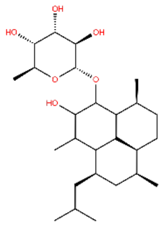	-29.75
No. 27	No data	No data	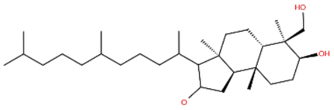	-33.27
No. 28	ZINC000044431210	474.59	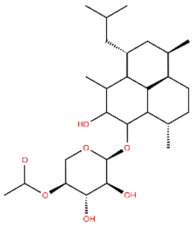	-32.83
No. 29	ZINC000006041800	476.60	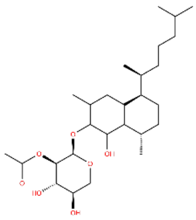	-32.97
No. 30	ZINC000040873606	412.61	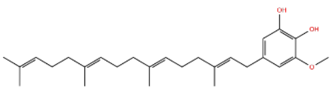	-37.87

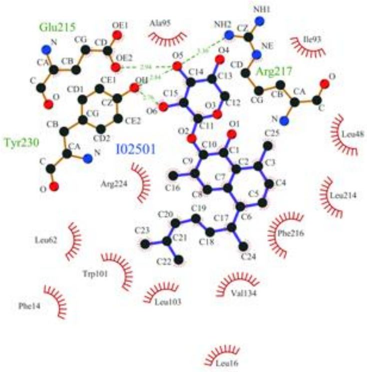

Inhibitor No.02

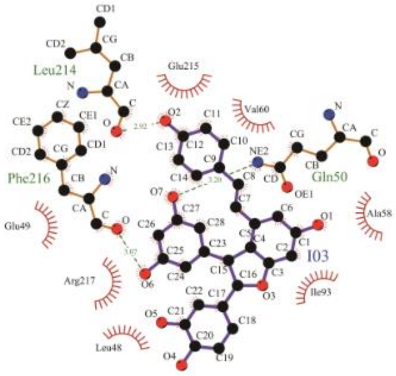

Inhibitor No.03

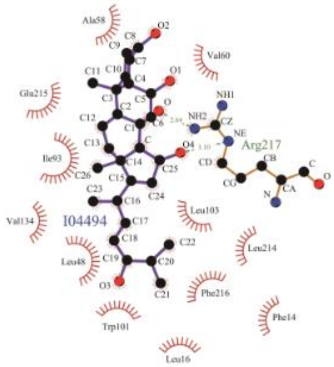

Inhibitor No.04

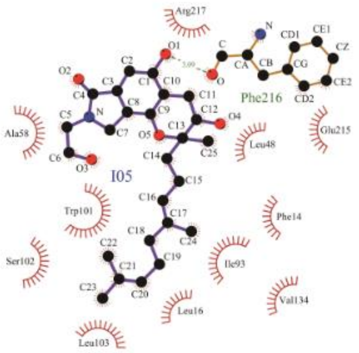

Inhibitor No.05

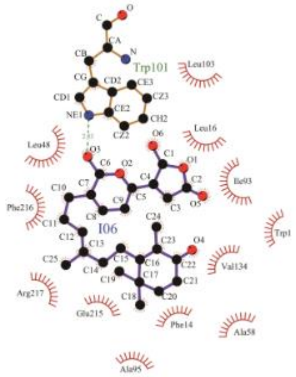

Inhibitor No.06

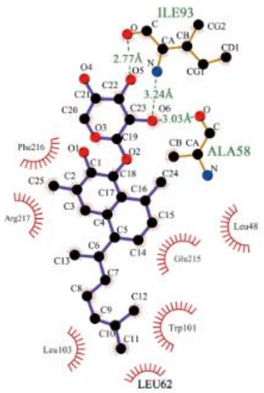

Inhibitor No.07

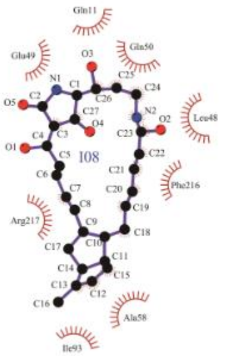

Inhibitor No.08

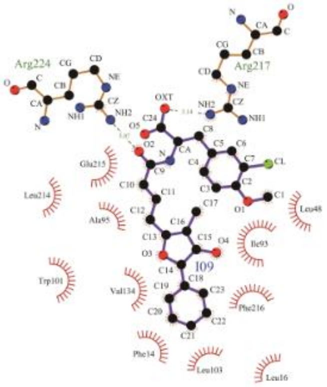

Inhibitor No.09

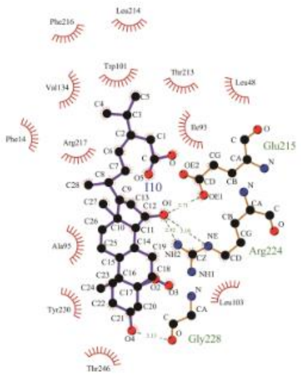

Inhibitor No.10

Figure representation

- Inhibitor bond
- Fascin residue bond
- Hydrogen bond and its leng
- Fascin residue involved in hydrophobic contact
- Corresponding atom involved in hydrophobic contact

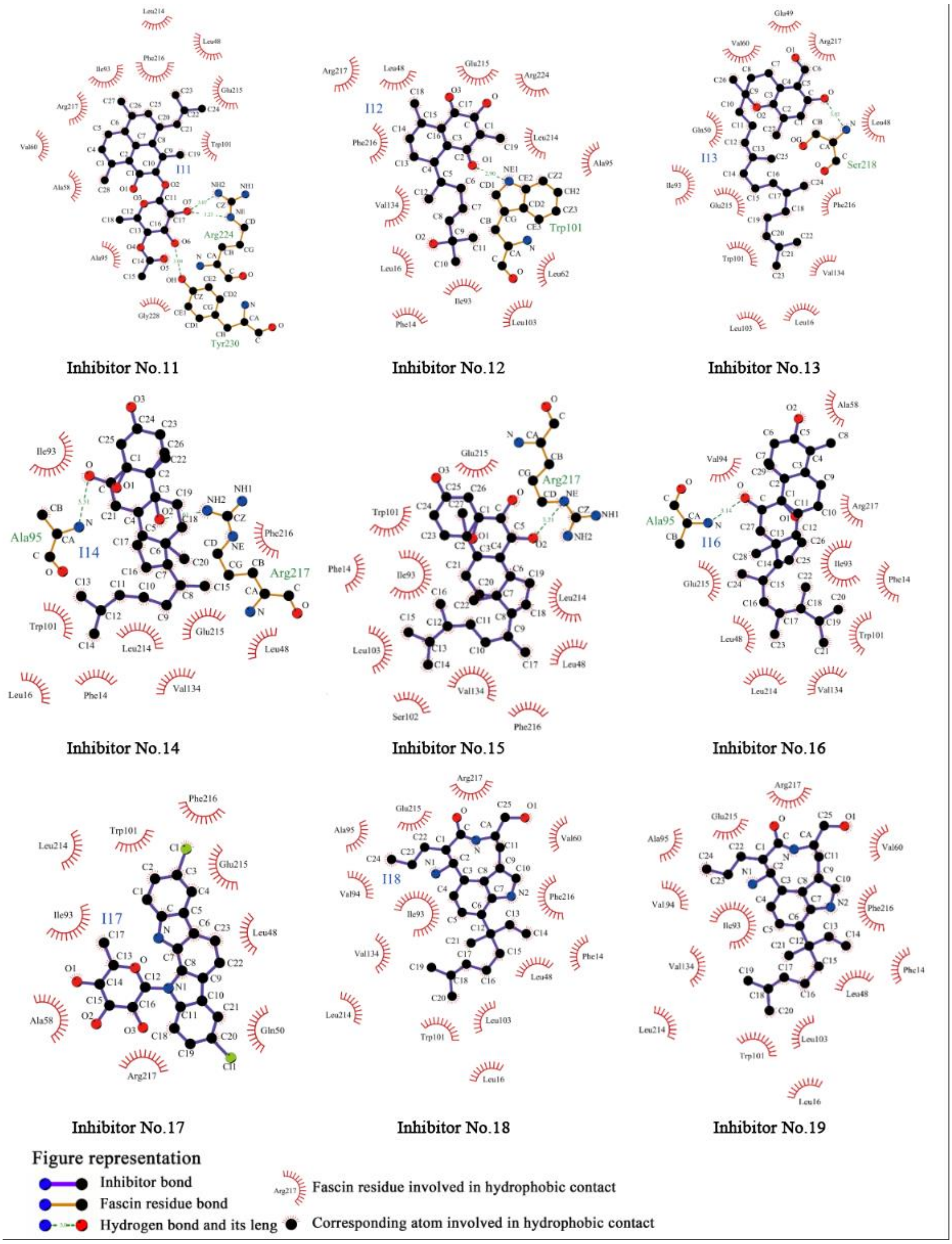

FIGURE S1 Interactions between Fascin and small-molecules No.2 to 19. (No.1 is the inhibitor NP-G2-029). Plotted by LIGPLOT Software (C. et al., 1995).

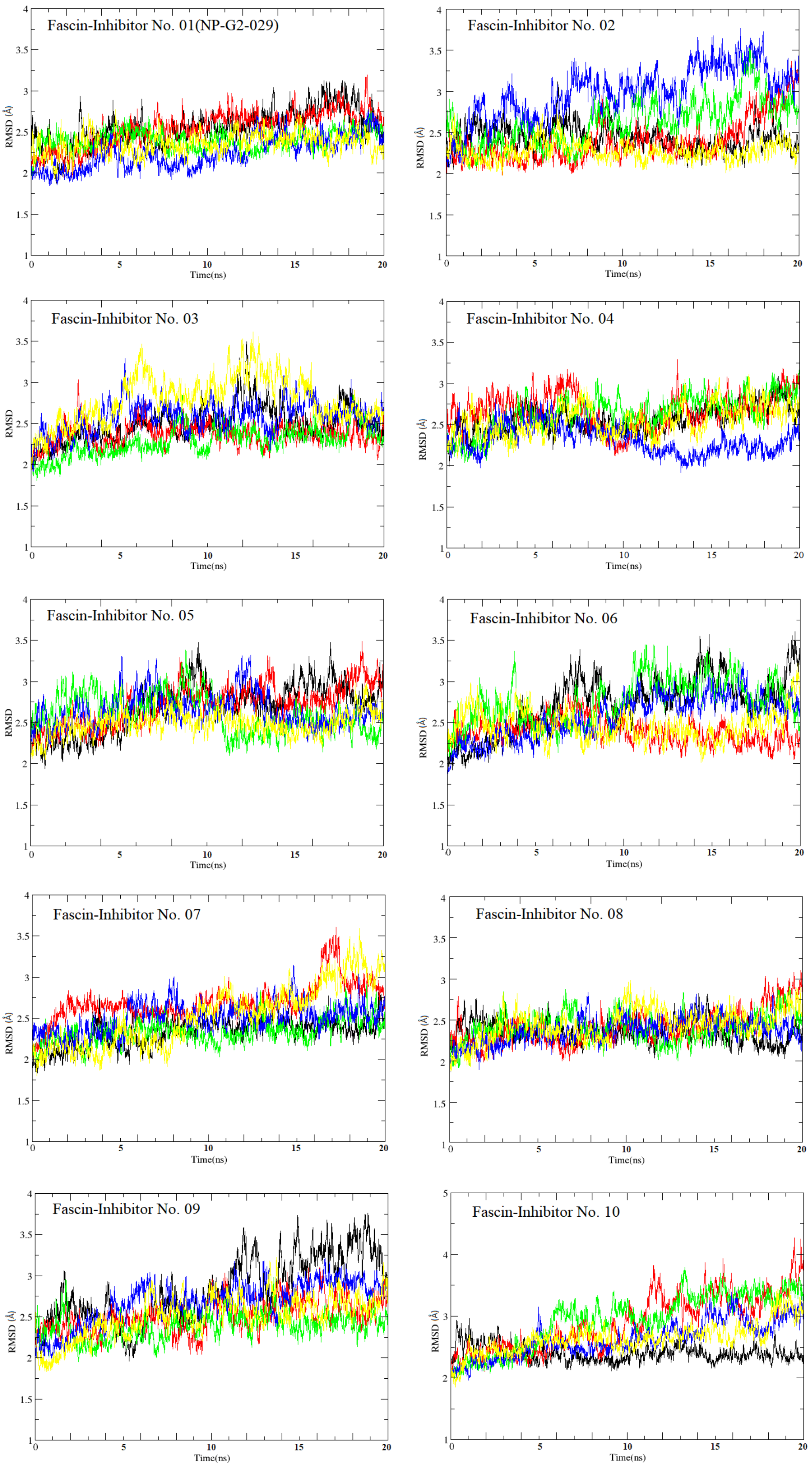

Fascin-Inhibitor No. 01(NP-G2-029)
RMSD (Å)
Time(ns)
Fascin-Inhibitor No. 02
RMSD (Å)
Time(ns)
Fascin-Inhibitor No. 03
RMSD
Time(ns)
Fascin-Inhibitor No. 04
RMSD (Å)
Time(ns)
Fascin-Inhibitor No. 05
RMSD
Time(ns)
Fascin-Inhibitor No. 06
RMSD (Å)
Time(ns)
Fascin-Inhibitor No. 07
RMSD (Å)
Time(ns)
Fascin-Inhibitor No. 08
RMSD (Å)
Time(ns)
Fascin-Inhibitor No. 09
RMSD (Å)
Time(ns)
Fascin-Inhibitor No. 10
RMSD (Å)
Time(ns)

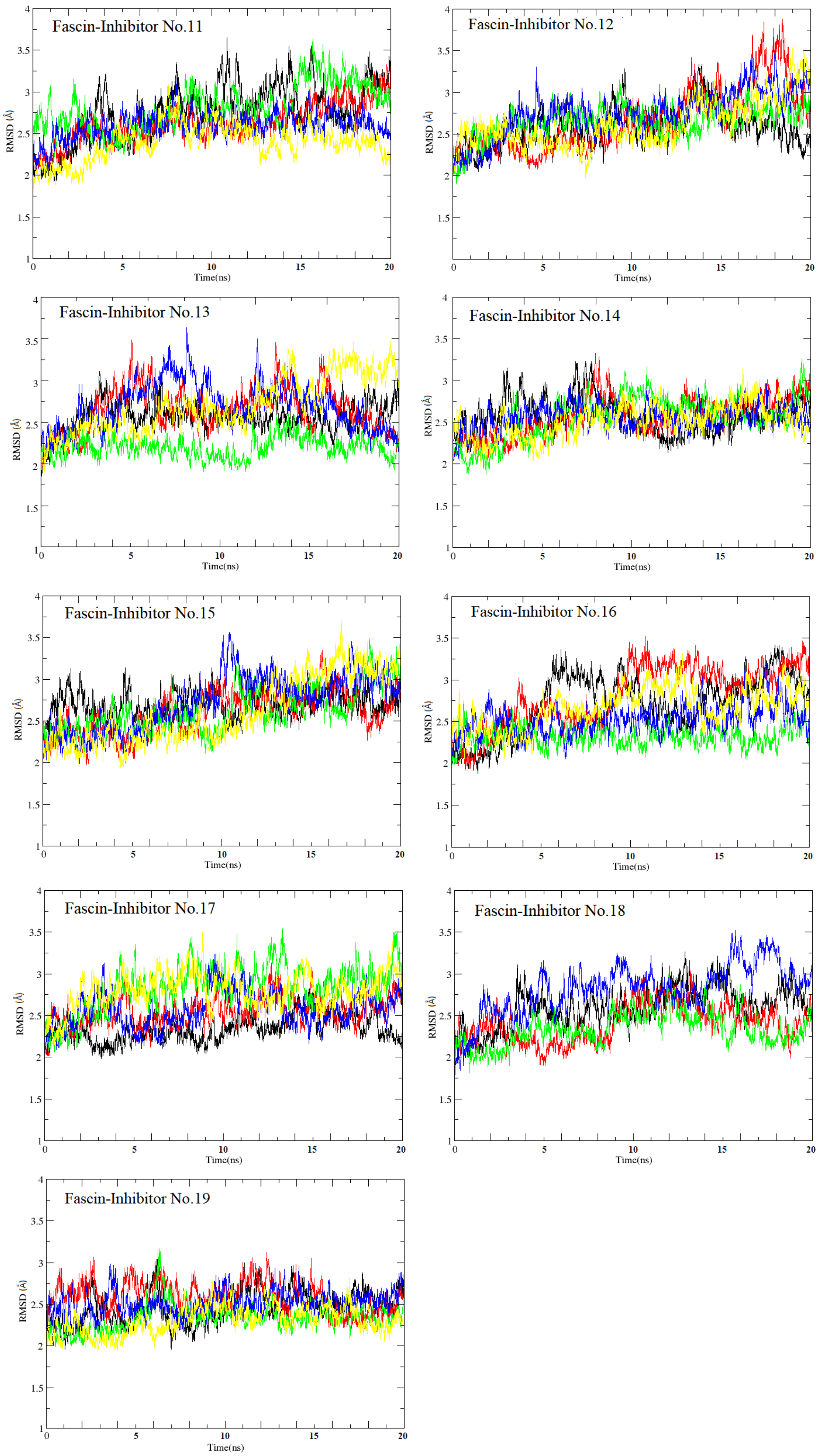
Fascin-Inhibitor No.11
Fascin-Inhibitor No.12
Fascin-Inhibitor No.13
Fascin-Inhibitor No.14
Fascin-Inhibitor No.15
Fascin-Inhibitor No.16
Fascin-Inhibitor No.17
Fascin-Inhibitor No.18
Fascin-Inhibitor No.19
RMSD (Å)
Time(ns)
1
1.5
2
2.5
3
3.5
4
0
5
10
15
20

FIGURE S2 RMSD files for each complex of Fascin with inhibitor No. 01-19. Fine color lines stand for 5 replicates of conventional MD simulation. RMSD calculated by using the CPPTRAJ module in AMBER 20 (Case et al., 2020).

[NP-G2-029]

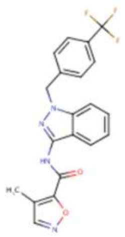

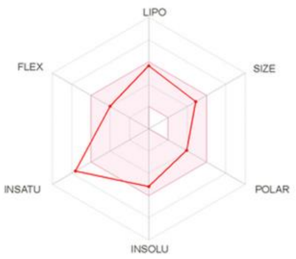

SMILES O=C(c1oncc1C)Nc1nn(c2c1cccc2)Cc1ccc(cc1)C(F)(F)F

Physicochemical Properties	
Formula	C20H15F3N4O2
Molecular weight	400.35 g/mol
Num. heavy atoms	29
Num. arom. heavy atoms	20
Fraction Csp3	0.15
Num. rotatable bonds	6
Num. H-bond acceptors	7
Num. H-bond donors	1
Molar Refractivity	99.73
TPSA	72.95 Å²

Lipophilicity	
Log $P_{o/w}$ (iLOGP)	2.62
Log $P_{o/w}$ (XLOGP3)	4.36
Log $P_{o/w}$ (WLOGP)	5.61
Log $P_{o/w}$ (MLOGP)	3.79
Log $P_{o/w}$ (SILICOS-IT)	3.90
Consensus Log $P_{o/w}$	4.05

Water Solubility	
Log *S* (ESOL)	-5.18
Solubility	2.62e-03 mg/ml ; 6.56e-06 mol/l
Class	Moderately soluble
Log *S* (Ali)	-5.61
Solubility	9.88e-04 mg/ml ; 2.47e-06 mol/l
Class	Moderately soluble
Log *S* (SILICOS-IT)	-7.56
Solubility	1.10e-05 mg/ml ; 2.74e-08 mol/l
Class	Poorly soluble

Pharmacokinetics	
GI absorption	High
BBB permeant	No
P-gp substrate	No
CYP1A2 inhibitor	Yes
CYP2C19 inhibitor	Yes
CYP2C9 inhibitor	Yes
CYP2D6 inhibitor	No
CYP3A4 inhibitor	No
Log K_p (skin permeation)	-5.65 cm/s

Druglikeness	
Lipinski	Yes; 0 violation
Ghose	No; 1 violation: WLOGP>5.6
Veber	Yes
Egan	Yes
Muegge	Yes
Bioavailability Score	0.55

Medicinal Chemistry	
PAINS	0 alert
Brenk	0 alert
Leadlikeness	No; 2 violations: MW>350, XLOGP3>3.5
Synthetic accessibility	3.08

[7]

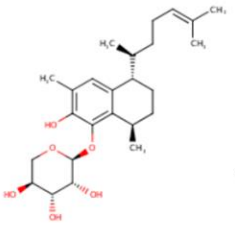

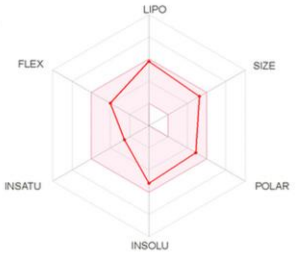

SMILES CC(=CCC[C@@H]([C@@H]1CC[C@H](c2c1cc(C)c(c2O[C@@H]1OC[C@@H]([C@H]([C@H]1O)O)O)C)C)C

Physicochemical Properties	
Formula	C25H38O6
Molecular weight	434.57 g/mol
Num. heavy atoms	31
Num. arom. heavy atoms	6
Fraction Csp3	0.68
Num. rotatable bonds	6
Num. H-bond acceptors	6
Num. H-bond donors	4
Molar Refractivity	121.83
TPSA	99.38 Å²

Lipophilicity	
Log $P_{o/w}$ (iLOGP)	3.85
Log $P_{o/w}$ (XLOGP3)	4.63
Log $P_{o/w}$ (WLOGP)	3.88
Log $P_{o/w}$ (MLOGP)	2.28
Log $P_{o/w}$ (SILICOS-IT)	3.66
Consensus Log $P_{o/w}$	3.66

Water Solubility	
Log *S* (ESOL)	-5.20
Solubility	2.75e-03 mg/ml ; 6.33e-06 mol/l
Class	Moderately soluble
Log *S* (Ali)	-6.44
Solubility	1.57e-04 mg/ml ; 3.61e-07 mol/l
Class	Poorly soluble
Log *S* (SILICOS-IT)	-3.47
Solubility	1.48e-01 mg/ml ; 3.41e-04 mol/l
Class	Soluble

Pharmacokinetics	
GI absorption	High
BBB permeant	No
P-gp substrate	Yes
CYP1A2 inhibitor	No
CYP2C19 inhibitor	No
CYP2C9 inhibitor	No
CYP2D6 inhibitor	Yes
CYP3A4 inhibitor	No
Log K_p (skin permeation)	-5.66 cm/s

Druglikeness	
Lipinski	Yes; 0 violation
Ghose	Yes
Veber	Yes
Egan	Yes
Muegge	Yes
Bioavailability Score	0.55

Medicinal Chemistry	
PAINS	0 alert
Brenk	1 alert: isolated_alkene
Leadlikeness	No; 2 violations: MW>350, XLOGP3>3.5
Synthetic accessibility	5.68

[15]

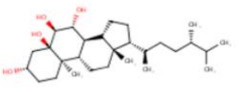

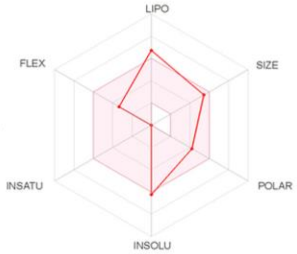

SMILES: O[C@H]1CC[C@]2([C@@](C1)(O)[C@@H](O)[C@@H]([C@@H]1[C@@H]2CC[C@@]2([C@H]1CC[C@@H]2[C@@H](CC[C@@H](C(C)C)C)C)C)O)C

Physicochemical Properties	
Formula	C28H50O4
Molecular weight	450.69 g/mol
Num. heavy atoms	32
Num. arom. heavy atoms	0
Fraction Csp3	1.00
Num. rotatable bonds	5
Num. H-bond acceptors	4
Num. H-bond donors	4
Molar Refractivity	132.42
TPSA	80.92 Å²

Lipophilicity	
Log $P_{o/w}$ (iLOGP)	3.15
Log $P_{o/w}$ (XLOGP3)	6.24
Log $P_{o/w}$ (WLOGP)	4.77
Log $P_{o/w}$ (MLOGP)	4.02
Log $P_{o/w}$ (SILICOS-IT)	4.38
Consensus Log $P_{o/w}$	4.51

Water Solubility	
Log *S* (ESOL)	-6.24
Solubility	2.62e-04 mg/ml ; 5.81e-07 mol/l
Class	Poorly soluble
Log *S* (Ali)	-7.73
Solubility	8.47e-06 mg/ml ; 1.88e-08 mol/l
Class	Poorly soluble
Log *S* (SILICOS-IT)	-3.80
Solubility	7.14e-02 mg/ml ; 1.58e-04 mol/l
Class	Soluble

Pharmacokinetics	
GI absorption	High
BBB permeant	No
P-gp substrate	Yes
CYP1A2 inhibitor	No
CYP2C19 inhibitor	No
CYP2C9 inhibitor	No
CYP2D6 inhibitor	No
CYP3A4 inhibitor	No
Log K_p (skin permeation)	-4.62 cm/s

Druglikeness	
Lipinski	Yes; 0 violation
Ghose	No; 2 violations: MR>130, #atoms>70
Veber	Yes
Egan	Yes
Muegge	No; 1 violation: XLOGP3>5
Bioavailability Score	0.55

Medicinal Chemistry	
PAINS	0 alert
Brenk	0 alert
Leadlikeness	No; 2 violations: MW>350, XLOGP3>3.5
Synthetic accessibility	6.23

[18]

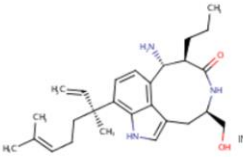

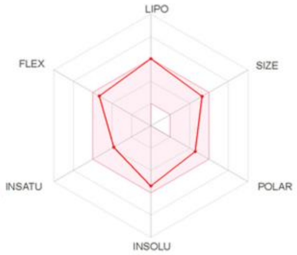

SMILES: CCC[C@H]1C(=O)N[C@@H](CO)Cc2c3c([C@@H]1N)ccc(c3[nH]c2)[C@@](CCC=C(C)C)(C=C)C

Physicochemical Properties	
Formula	C27H39N3O2
Molecular weight	437.62 g/mol
Num. heavy atoms	32
Num. arom. heavy atoms	9
Fraction Csp3	0.52
Num. rotatable bonds	8
Num. H-bond acceptors	3
Num. H-bond donors	4
Molar Refractivity	137.72
TPSA	91.14 Å²

Lipophilicity	
Log $P_{o/w}$ (iLOGP)	3.54
Log $P_{o/w}$ (XLOGP3)	4.99
Log $P_{o/w}$ (WLOGP)	4.10
Log $P_{o/w}$ (MLOGP)	2.90
Log $P_{o/w}$ (SILICOS-IT)	5.68
Consensus Log $P_{o/w}$	4.24

Water Solubility	
Log *S* (ESOL)	-5.38
Solubility	1.84e-03 mg/ml ; 4.20e-06 mol/l
Class	Moderately soluble
Log *S* (Ali)	-6.64
Solubility	9.95e-05 mg/ml ; 2.27e-07 mol/l
Class	Poorly soluble
Log *S* (SILICOS-IT)	-7.03
Solubility	4.07e-05 mg/ml ; 9.31e-08 mol/l
Class	Poorly soluble

Pharmacokinetics	
GI absorption	High
BBB permeant	No
P-gp substrate	Yes
CYP1A2 inhibitor	No
CYP2C19 inhibitor	No
CYP2C9 inhibitor	No
CYP2D6 inhibitor	No
CYP3A4 inhibitor	Yes
Log K_p (skin permeation)	-5.43 cm/s

Druglikeness	
Lipinski	Yes; 0 violation
Ghose	No; 2 violations: MR>130, #atoms>70
Veber	Yes
Egan	Yes
Muegge	Yes
Bioavailability Score	0.55

Medicinal Chemistry	
PAINS	0 alert
Brenk	1 alert: isolated_alkene
Leadlikeness	No; 3 violations: MW>350, Rotors>7, XLOGP3>3.5
Synthetic accessibility	5.18

FIGURE S3 Toxicity data for potential inhibitor No. 07, 15, 18. Evaluation processed by ProTox-II server (Banerjee et al., 2018).

TABLE S4 Toxicity evaluation data collection. Evaluation processed by ProTox-II server (Banerjee et al., 2018).

ITEM		NP-G2-029		I07		I15		I018	
**Classification**	**Target**	**Prediction**	**Probability**	**Prediction**	**Probability**	**Prediction**	**Probability**	**Prediction**	**Probability**
Organ toxicity	Hepatotoxicity	Active	0.52	Inactive	0.91	Inactive	0.84	Inactive	0.72
Toxicity end points	Carcinogenicity	Active	0.58	Inactive	0.65	Inactive	0.58	Inactive	0.63
Toxicity end points	Immunotoxicity	Inactive	0.99	Active	0.99	Active	0.99	Active	0.99
Toxicity end points	Mutagenicity	Active	0.6	Inactive	0.82	Active	0.64	Inactive	0.68
Toxicity end points	Cytotoxicity	Inactive	0.75	Inactive	0.82	Inactive	0.98	Inactive	0.73
Tox21-Nuclear receptor signalling pathways	Aryl hydrocarbon Receptor	Inactive	0.62	Inactive	0.86	Inactive	0.99	Inactive	0.79
Tox21-Nuclear receptor signalling pathways	Androgen Receptor	Inactive	0.97	Inactive	0.97	Inactive	0.95	Inactive	0.93
Tox21-Nuclear receptor signalling pathways	Androgen Receptor Ligand Binding Domain	Inactive	0.96	Inactive	0.93	Inactive	0.95	Inactive	0.98
Tox21-Nuclear receptor signalling pathways	Aromatase	Inactive	0.84	Inactive	0.59	Inactive	0.94	Inactive	0.9
Tox21-Nuclear receptor signalling pathways	Estrogen Receptor Alpha (ER)	Inactive	0.82	Inactive	0.79	Inactive	0.74	Inactive	0.89
Tox21-Nuclear receptor signalling pathways	Estrogen Receptor Ligand Binding Domain (ER-LBD)	Inactive	0.91	Inactive	0.92	Inactive	0.86	Inactive	0.96
Tox21-Nuclear receptor signalling pathways	Peroxisome Proliferator Activated Receptor Gamma (PPAR-Gamma)	Inactive	0.91	Inactive	0.95	Inactive	0.99	Inactive	0.91
Tox21-Stress response pathways	Nuclear factor (erythroid-derived 2)-like 2/antioxidant responsive element	Inactive	0.94	Inactive	0.85	Inactive	0.84	Inactive	0.92
Tox21-Stress response pathways	Heat shock factor response element	Inactive	0.94	Inactive	0.85	Inactive	0.84	Inactive	0.92
Tox21-Stress response pathways	Mitochondrial Membrane Potential	Inactive	0.62	Inactive	0.54	Active	0.52	Inactive	0.8
Tox21-Stress response pathways	Phosphoprotein (Tumor Supressor) p53	Inactive	0.86	Inactive	0.64	Inactive	0.78	Inactive	0.82
Tox21-Stress response pathways	ATPase family AAA domain-containing protein 5 (ATAD5)	Inactive	0.85	Inactive	0.92	Inactive	0.91	Inactive	0.88

Reference

Banerjee, P., Eckert, A. O., Schrey, A. K., and Preissner, R. (2018). ProTox-II: a webserver for the prediction of toxicity of chemicals, *Nucleic Acids Res.* 46: W257-W63

C., A., Wallace, A., R., Laskowski, and Thornton, J. M. (1995). LIGPLOT: a program to generate schematic diagrams of protein-ligand interactions, *Protein Eng.* 8: 127-34

Case, D. A., K. Belfon, I.Y. Ben-Shalom, S.R. Brozell, D.S. Cerutti, T.E. Cheatham, III, V. W. D. C., T.A. Darden, R.E. Duke, G. G., M.K. Gilson, H. Gohlke, A.W. Goetz, R Harris, S. I., S.A. Iz, m., K. Kasavajhala, A. Kovalenko, R. Krasny, T. Kurtzman, T.S. Lee, S. L., P. Li, and C. Lin, J. L. (2020). AMBER 2020, *University of California, San Francisco*
