# Supplementary material for: Potential Inhibitors of Fascin From A Database of Marine Natural Products: A Virtual Screening and Molecular Dynamics Study
Source: Front Chem. 2021 Oct 7;9:719949. doi: 10.3389/fchem.2021.719949 (PMC8529705; doi:10.3389/fchem.2021.719949)
Supplement: Supplementary file 2 [file DataSheet1.ZIP › Raw Date for Manuscript-719949-reduced/Figure 2/Figure 2. The workflow of inhibitors screening in this study.pptx]

## Slide 1
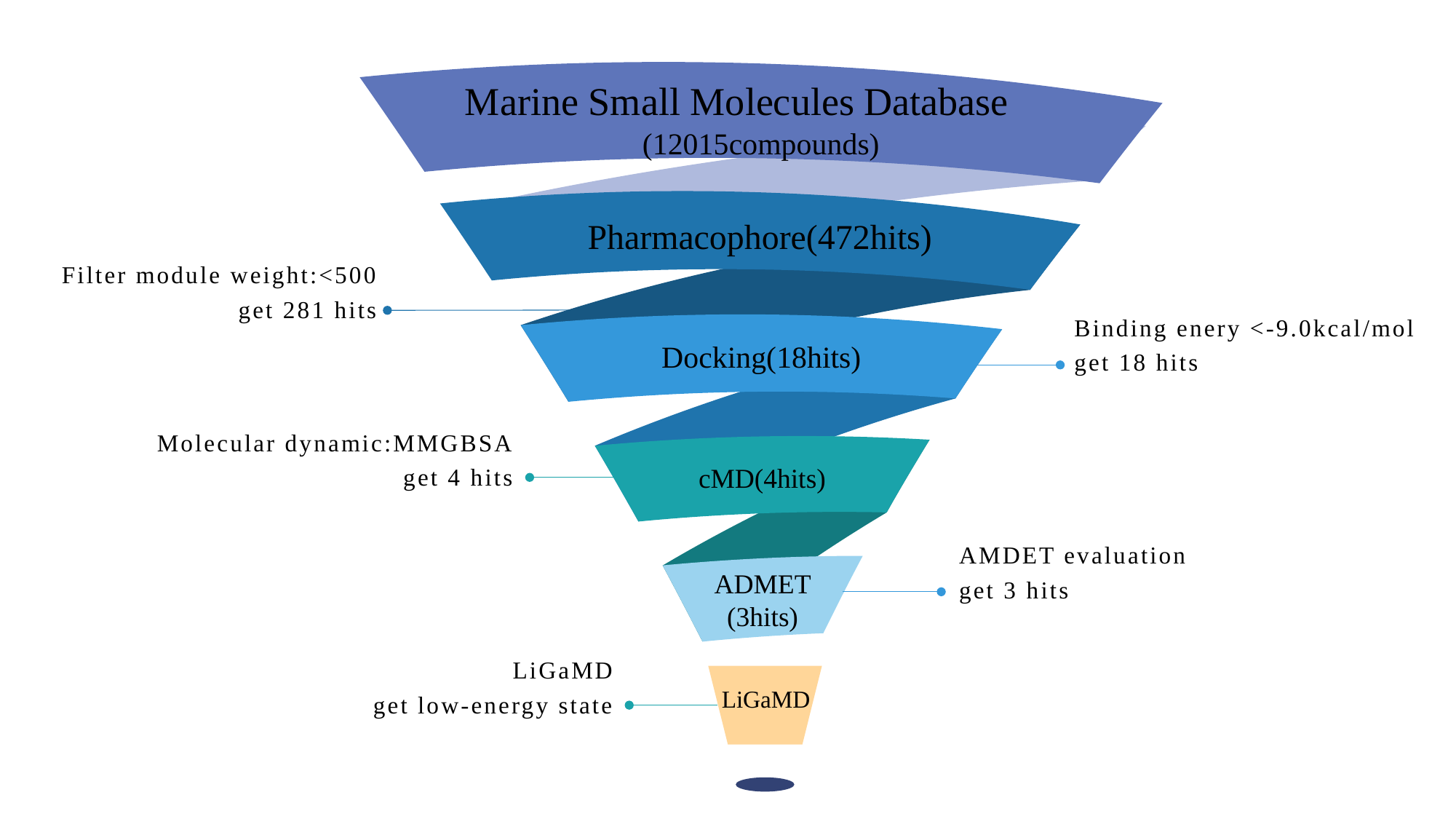

Marine Small Molecules Database
(12015compounds)
Pharmacophore(472hits)
Filter module weight:<500
get 281 hits
Binding enery <-9.0kcal/mol
get 18 hits
Docking(18hits)
Molecular dynamic:MMGBSA
get 4 hits
cMD(4hits)
AMDET evaluation
get 3 hits
ADMET
(3hits)
LiGaMD
get low-energy state
LiGaMD
